# Decoupling of Tree‐Ring Cellulose *δ* ^18^O and *δ* ^2^H Highlighted by Their Contrasting Relationships to Climate and Tree Intrinsic Variables

**DOI:** 10.1111/pce.15252

**Published:** 2024-11-07

**Authors:** Justine Charlet de Sauvage, Matthias Saurer, Kerstin Treydte, Mathieu Lévesque

**Affiliations:** ^1^ Silviculture Group, Institute of Terrestrial Ecosystems, ETH Zurich Zurich Switzerland; ^2^ Forest Dynamics, Swiss Federal Institute for Forest, Snow and Landscape Research WSL Birmensdorf Switzerland; ^3^ Oeschger Centre for Climate Change Research University of Bern Bern Switzerland

**Keywords:** *Abies alba*, *Pseudotsuga menziesii*, stable isotope, tree age, tree crown, tree‐ring width, vapour pressure deficit

## Abstract

Oxygen (*δ* ^18^O) and hydrogen (*δ* ^2^H) stable isotope ratios are tightly coupled in precipitation and, albeit damped, in leaf water, but are often decoupled in tree‐ring cellulose. The environmental and physiological conditions in which this decoupling occurs are not yet well understood. We investigated the relationships between *δ* ^18^O and *δ* ^2^H and tree‐ring width (TRW), tree crown volume, tree age and climate in silver fir and Douglas‐fir and found substantial differences between *δ* ^18^O and *δ* ^2^H. Overall, *δ* ^18^O–*δ* ^2^H correlations were weak to absent but became significantly negative under high summer vapour pressure deficit (VPD). *δ* ^18^O and *δ* ^2^H had positive and negative nonlinear relationships with TRW, respectively, with clear relationships at the site and tree levels for silver fir and, to a lesser extent, for Douglas‐fir. Age trends for silver fir were weakly negative in *δ* ^18^O but positive in *δ* ^2^H. Tree crown volume and *δ* ^18^O or *δ* ^2^H had no significant relationships. Most strikingly, *δ* ^18^O strongly depended on spring climate (precipitation and VPD), whereas *δ* ^2^H depended on summer climate (temperature and VPD) for both species. Our study shows that the *δ* ^18^O–*δ* ^2^H decoupling in tree‐ring cellulose in two temperate conifer species could be highlighted by their contrasting relationships to climate and tree intrinsic variables (TRW, age).

## Summary statement

In tree‐ring cellulose of silver fir (*Abies alba* Mill.) and Douglas‐fir (*Pseudotsuga menziesii* (Mirb.) Franco), we observed a decoupling of *δ* ^18^O and *δ* ^2^H and highlighted it by the contrasting relationships of *δ* ^18^O and *δ* ^2^H to climate and to tree‐ring width or to a lesser extent tree age.

## 1 Introduction

Stable oxygen and hydrogen isotope ratios (*δ* ^18^O and *δ* ^2^H) in precipitation are highly variable across the globe (Cernusak et al. 2016). Nevertheless, a strong linear relationship exists between *δ* ^18^O and *δ* ^2^H in water from precipitation, known as the global meteoric water line (Craig 1961). Plants take up water (H_2_O) mainly from the soil and carbon dioxide (CO_2_) from the air as reactants for photosynthesis to produce carbohydrates (C_6_H_12_O_6_) and dioxygen (O_2_). Before photosynthesis, the *δ* ^18^O in the leaf water becomes ^18^O‐enriched in comparison to soil water due to stronger evaporation from the leaf of the lighter isotope ^16^O compared to ^18^O (Dongmann et al. 1974). In leaf water, *δ* ^18^O and *δ* ^2^H are still highly correlated but already depart from the meteoric water line (Cernusak et al. 2022). DeNiro and Epstein (1979) reported a complete exchange of oxygen atoms between H_2_O and CO_2_ before photosynthesis, transferring the isotope signal from the leaf water to the CO_2_. Therefore, *δ* ^18^O and *δ* ^2^H variations in leaf sugars originate from the leaf water and would be expected to still correlate with each other after photosynthesis. However, post‐photosynthetic isotope fractionation processes may alter the *δ* ^18^O–*δ* ^2^H relationship. The *δ* ^18^O–*δ* ^2^H relationship was reported to lose strength in the leaf cellulose and branch wood cellulose (Holloway‐Phillips et al. 2023) and *δ* ^2^H in leaf cellulose highly depends on *δ* ^2^H variations in leaf sucrose (Holloway‐Phillips et al. 2022).

Several studies have also reported a weak or absent *δ* ^18^O–*δ* ^2^H correlation in tree‐ring cellulose (e.g., Lehmann et al. 2022; Nabeshima et al. 2018 [in earlywood but not in latewood]; Vitali et al. 2022). It is known that during tree‐ring formation, the newly synthesized cellulose not only carries a leaf water signal, passed on via leaf sugars, but also a source water signal. Mixing of different carbohydrate pools and biochemical processes during sink cell processes may modify the isotope ratios (Roden, Lin, and Ehleringer 2000). Nevertheless, the weak *δ* ^18^O–*δ* ^2^H correlation in tree‐ring cellulose remains largely unexplained, partly because of a still limited understanding of the processes driving *δ* ^2^H (Lehmann et al. 2022). A better understanding of the factors influencing the tree‐ring cellulose *δ* ^18^O–*δ* ^2^H relationship has the potential to add valuable knowledge on the different physiological processes shaping *δ* ^18^O and *δ* ^2^H values, from photosynthesis to tree‐ring cellulose formation.

In tree‐ring cellulose *δ* ^18^O, the source water *δ* ^18^O signal comes from the exchange of oxygen atoms between xylem water and the carbonyl groups of the carbohydrates transported in the phloem, with the isotope exchange rate being highly variable (Martínez‐Sancho et al. 2023; Sternberg et al. 2006). Tree‐ring *δ* ^18^O is a commonly used proxy for reconstructing past climatic conditions because of its sensitivity to air temperature, precipitation sum and air dryness, even at temperate sites where temperature and precipitation do not limit growth (Hartl‐Meier et al. 2015; Saurer et al. 2008; Treydte et al. 2024). However, little is known about the effects of the intrinsic characteristics of a tree, such as age or crown volume, on tree‐ring cellulose *δ* ^18^O (but see e.g., Klesse et al. 2018). This would also help for understanding the *δ* ^18^O–*δ* ^2^H relationship. Further, the relationship between tree‐ring *δ* ^18^O and tree‐ring width (TRW) has not yet been analysed in detail, and it is unclear if it follows a general pattern across species. For example, Lévesque et al. (2013) found some significant negative correlations between *δ* ^18^O in earlywood and earlywood width and some significant positive correlations between *δ* ^18^O in latewood and latewood width, but not in all conifer species analysed.

*δ* ^2^H in tree‐ring cellulose has been increasingly used in recent years with the help of more accessible measurement techniques (Lehmann et al. 2022). Like *δ* ^18^O, it is expected to carry a source water signal (Dawson et al. 2002). During cellulose synthesis, carbon‐bound hydrogen atoms – the atoms that are eventually measured in tree‐ring cellulose *δ* ^2^H – also undergo post‐photosynthetic isotope fractionation processes catalysed by specific enzymes (Augusti, Betson, and Schleucher 2006, 2008). Recent studies suggested that *δ* ^2^H in tree‐ring cellulose carries a strong physiological signal and a weaker climatic signal than carbon isotope ratios (*δ* ^13^C) or *δ* ^18^O (Lehmann et al. 2021; Vitali et al. 2022, 2023). Analysing if the *δ* ^18^O–*δ* ^2^H relationship varies with climatic conditions could bring valuable knowledge on both isotope ratios and their covariation. Recently, two studies reported a negative relationship between *δ* ^2^H and TRW, but the results were not consistent across all sites (Lehmann et al. 2021; Vitali et al. 2022). An increase in *δ* ^2^H values in tree‐ring cellulose could also indicate a higher reliance on ^2^H‐enriched stored carbohydrates (Lehmann et al. 2021). Since physiological signals have been suggested to be imprinted in tree‐ring cellulose *δ* ^2^H (Lehmann et al. 2022), tree intrinsic parameters such as age or crown volume might affect *δ* ^2^H but have been poorly studied (but see for tree age, e.g., Nakatsuka et al. 2020).

Our study aims to highlight the often‐observed decoupling between *δ* ^18^O and *δ* ^2^H by using tree‐ring isotope data of silver fir (*Abies alba* Mill.) and Douglas‐fir (*Pseudotsuga menziesii* (Mirb.) Franco) growing in three temperate forests in Switzerland. We analysed the *δ* ^18^O–*δ* ^2^H relationship in tree‐ring cellulose and investigated the relationships of each of the two variables with the intrinsic characteristics of a tree and with climatic variables. Specifically, we asked the following questions: (1) What is the *δ* ^18^O–*δ* ^2^H relationship in tree‐ring cellulose of silver fir and Douglas‐fir at the tree and site levels? (2) What are the relationships between tree‐ring stable isotope ratios and TRW, tree crown volume and tree age? (3) What are the responses of *δ* ^18^O, *δ* ^2^H and the *δ* ^18^O–*δ* ^2^H relationship to climate variability?

## 2 Materials and Methods

### 2.1 Species and Site Selection

**Figure 1:**
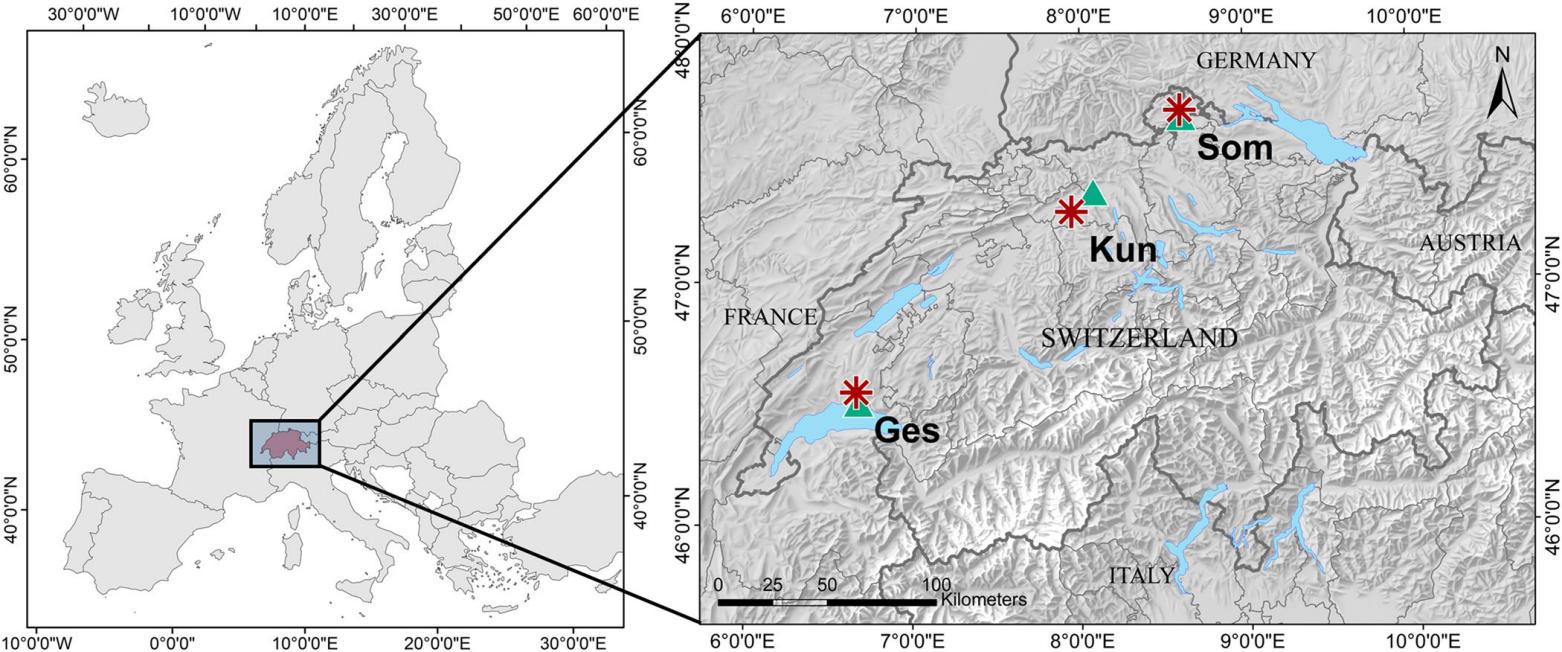
Location of the study sites within Europe (left, © EuroGeographics for the administrative boundaries) and Switzerland (right). The red stars indicate the study sites (the full names are given in Table 1), and the green triangles show the location of the meteorological stations used for the analyses. The right background map is from the Federal Office of Topography swisstopo. The geographical coordinates of the sites and meteorological stations are provided in Table S1.

For this study, we selected one native and one non‐native conifer species at three sites in the lowlands of Switzerland (Figure 1 and Table S1). These two species are of interest in Central European forestry because of their timber quality and are possible alternatives to Norway spruce (*Picea abies* (L.) H. Karst.). Silver fir is a species native to Europe and mostly growing in mountainous regions apart from Northern Europe (Wolf 2003). The species is considered to be more resistant to drought than Norway spruce (van der Maaten‐Theunissen, Kahle, and van der Maaten 2013) and potentially able to cope with a future warmer and drier climate (Vitasse et al. 2019). Douglas‐fir is a non‐native species originating from western North America that was introduced in the first half of the 19th century in Central Europe (Spiecker, Lindner, and Schuler 2019). This species has also been shown to tolerate droughts relatively well (Lévesque et al. 2014; Vitali, Büntgen, and Bauhus 2017). However, the physiological responses of these species to climate change are still not fully understood and have not been extensively studied based on stable isotope ratios from tree‐ring cellulose.

Both species are present at all three selected sites. The sites are located between 480 and 760 m a.s.l. and present temperate climatic conditions with a mean annual temperature between 9.5°C and 9.9°C and a mean annual precipitation sum between 917 and 1094 mm for the period 2000–2020 (Table S1 and Figure S1). The terrain at the three study sites is flat, with brown soil, and the stands are essentially composed of silver fir, Norway spruce, Douglas‐fir, European beech (*Fagus sylvatica* L.) and sycamore maple (*Acer pseudoplatanus* L.), with some other less abundant species.

### 2.2 Data Collection and Tree‐Ring Width Measurements

At each site and for each species, eight healthy and dominant or codominant silver fir and Douglas‐fir trees were sampled between the end of August 2020 and May 2021. Trees were selected to have similar diameters at breast height (DBH) within a species at a given site (Table 1). To estimate the crown volume of the trees, eight radii of the crown were measured with a measuring tape following the eight cardinal and intercardinal directions. The total tree height and height of the crown base were also measured with a Vertex 4 measuring device (Haglöf, Sweden). The crown volume was then calculated assuming that it had the shape of an octagonal pyramid with an irregular base. In addition, the DBH of the trees was measured.

**Table 1:** Characteristics of the sampled trees. The full names of the sites are given together with the three‐letter code used elsewhere in the text and figures. DBH (Diameter at Breast Height), tree height, estimated age at coring height, and TRW (tree‐ring width of the period 2000–2020, i.e., the study period) are given with mean ± standard deviation of the eight trees of each species and at each site.

Species	Site	DBH (cm)	Tree height (m)	Est. age (years)	TRW (mm)
Silver fir	Bois des Gésiaux (Ges)	49.6 ± 3.4	26.7 ± 2.2	38 ± 9	6.45 ± 2.69
	Küngoldingen (Kun)	70.5 ± 7.9	37.2 ± 3.0	98 ± 16	3.05 ± 2.55
	Sommerwies (Som)	48.0 ± 13.5	33.3 ± 3.8	56 ± 16	5.05 ± 2.89
Douglas‐fir	Ges	104.5 ± 13.7	51.0 ± 2.8	104 ± 8	3.03 ± 1.69
	Kun	85.9 ± 12.9	49.8 ± 3.1	99 ± 9	4.13 ± 2.16
	Som	78.2 ± 14.9	45.9 ± 4.7	88 ± 17	3.76 ± 1.41

For dendrochronological analysis, two increment cores were extracted from each tree perpendicular to the slope with a 40‐ or 60‐cm long increment borer (5.15 mm core diameter; Häglof, Sweden). The cores were extracted at ca. 50 cm from the ground to minimize damage to the most economically valuable part of the tree. Increment cores were then air‐dried, glued on wooden holders and sanded. Tree‐ring widths were measured to the nearest 0.01 mm with a Lintab 5 measuring table connected to the software TSAP‐Win (v4.81; both from RINNTECH, Heidelberg, Germany) or based on scanned images (1200 dpi; Epson Expression 10000 XL, Japan Epson) with the software CooRecorder 9.6 and CDendro 9.6 (Cybis, Sweden). Both methods provided accurate and comparable measurements. Tree‐ring widths were visually crossdated with the measuring software and statistically checked with the software COFECHA (Holmes 1983). For crossdating, a total of 20 trees per site and species that were sampled in Charlet de Sauvage et al. (2023) were used. However, tree‐ring width data in this study included only the eight trees per site and species described in Table 1.

The age of each tree (hereafter referred to as *age*) was estimated at coring height based on the number of tree rings on the increment core with the highest number of tree rings in combination with the estimated number of missing rings to the pith. The pith‐offset estimation was based on the curvature of the rings, and the number of missing rings to the pith was approximated based on the distance to the pith and the width of the five innermost measured rings with the software CooRecorder 9.6 (Cybis, Sweden).

### 2.3 Stable Isotope Ratios in Tree‐Ring Cellulose

For each selected tree, we chose the core with the best quality (i.e., not broken, without missing rings and correctly crossdated) and with the highest correlation with the site chronology for isotopic analysis. We analysed each ring separately and did not pool the tree rings from two cores or from different trees. Further, we analysed whole rings instead of early‐ and latewood separately because the transition between earlywood and latewood is gradual in silver fir and Douglas‐fir and does not allow for accurate separation. Due to the presence of narrow rings, the use of the whole rings also ensured enough material for the cellulose extraction and isotopic measurements. All tree rings for the period 2000–2020 from 24 silver firs (8 trees × 3 sites) and 24 Douglas‐firs (8 trees × 3 sites) were analysed, resulting in a total of 1008 samples (8 trees × 3 sites × 2 species × 21 years).

To facilitate cellulose extraction, the individual tree rings were split into thin slices with a scalpel under a stereomicroscope. The wood samples were individually packed into fibre filter bags (F57, Ankom Technology, USA) for cellulose extraction following the procedure of Boettger et al. (2007), modified according to Weigt et al. (2015). The cellulose extracted yielded 51.6% ± 4.1% and 54.5% ± 3.1% (mean ± standard deviation) of the original wood mass of the tree rings for silver fir and Douglas‐fir, respectively. The cellulose samples were then homogenized with an ultrasonic device (UP200S, Hielscher Ultrasonics, Germany) in ca. 1 mL of distilled water, following Laumer et al. (2009). To remove the water left from homogenization, samples were freeze‐dried for up to 2 days (Beta 1‐8 LD plus, Christ, Germany). Then, 1 ± 0.05 mg of cellulose was packed in silver capsules (3.3 × 5 mm, Säntis Analytical, Switzerland).

To measure *δ* ^2^H and account for and correct for the exchangeable H in OH‐groups of cellulose, the samples were equilibrated with water of known isotopic composition following the methodology of Schuler et al. (2022). The equilibration system consisted of a heating oven, an equilibration chamber capable of holding a sample tray of up to 100 samples and a peristaltic pump (Gilson Incorporated, Middleton, USA). The chamber has inlet and outlet lines, where the inlet leads to the peristaltic pump (set at a flow of 1.7 mL/h) with a connection to a 50 mL falcon tube containing the equilibration water. The temperature of the oven was set to a constant temperature of 130°C, ensuring immediate evaporation of water after entering the equilibration chamber. After 2 h of equilibration, the chamber was flushed with dry nitrogen gas to ensure complete drying of the samples kept still at 130°C. The samples were then measured by thermal decomposition at 1420°C with a TC/EA (Elementar, Hanau, Germany) connected to an IRMS (MAT 253, Thermo). The *δ* ^2^H of carbon‐bound, non‐exchangeable H was subsequently calculated according to Schuler et al. (2022). All reported *δ* ^2^H values in the results refer to non‐exchangeable H. The precision of the analysis was 0.2‰ for *δ* ^18^O and 3‰ for *δ* ^2^H. Primary reference materials for *δ* ^2^H calibration were IAEA‐CH‐7 polyethylene foil (International Atomic Energy Agency, Vienna, Austria) and USGS62, USGS63 and USGS64 caffeine standards (United States Geological Survey, Reston, Virginia, USA), which were used to calibrate in‐house cellulose reference materials with a large range of isotope values. More details on the calibration are given in Schuler et al. (2022).

### 2.4 Climate Data

Hourly average air temperature, precipitation sum and relative humidity data and average daily global radiation data for the period 1999–2020 were retrieved from meteorological stations of MeteoSwiss located 3–13 km from the study sites (Figure 1 and Table S1). Temperature values were corrected for the differences in elevation between the sites and the meteorological stations, with published monthly temperature lapse rates from Lotter et al. (2002). We then used the daily minimum and maximum values of temperature and relative humidity to calculate the daily vapour pressure deficit (VPD; kPa), which indicates the actual evaporative capacity of the atmosphere (Allen et al. 1998) (for details see Equation S1). VPD directly affects photosynthesis, stomatal conductance, transpiration, water use or growth and is rising with climate change, which makes it interesting to study plant response to climate change (Novick et al. 2024).

To consider the influence of stable isotope ratios from local source water, monthly *δ* ^18^O and *δ* ^2^H values from precipitation were retrieved with the tool Piso.AI based on the geographical coordinates and elevation of the sites. The data provided is modelled using machine learning algorithms as described in Nelson, Basler and Kahmen (2021).

### 2.5 Statistical Analyses

**Figure 2:**
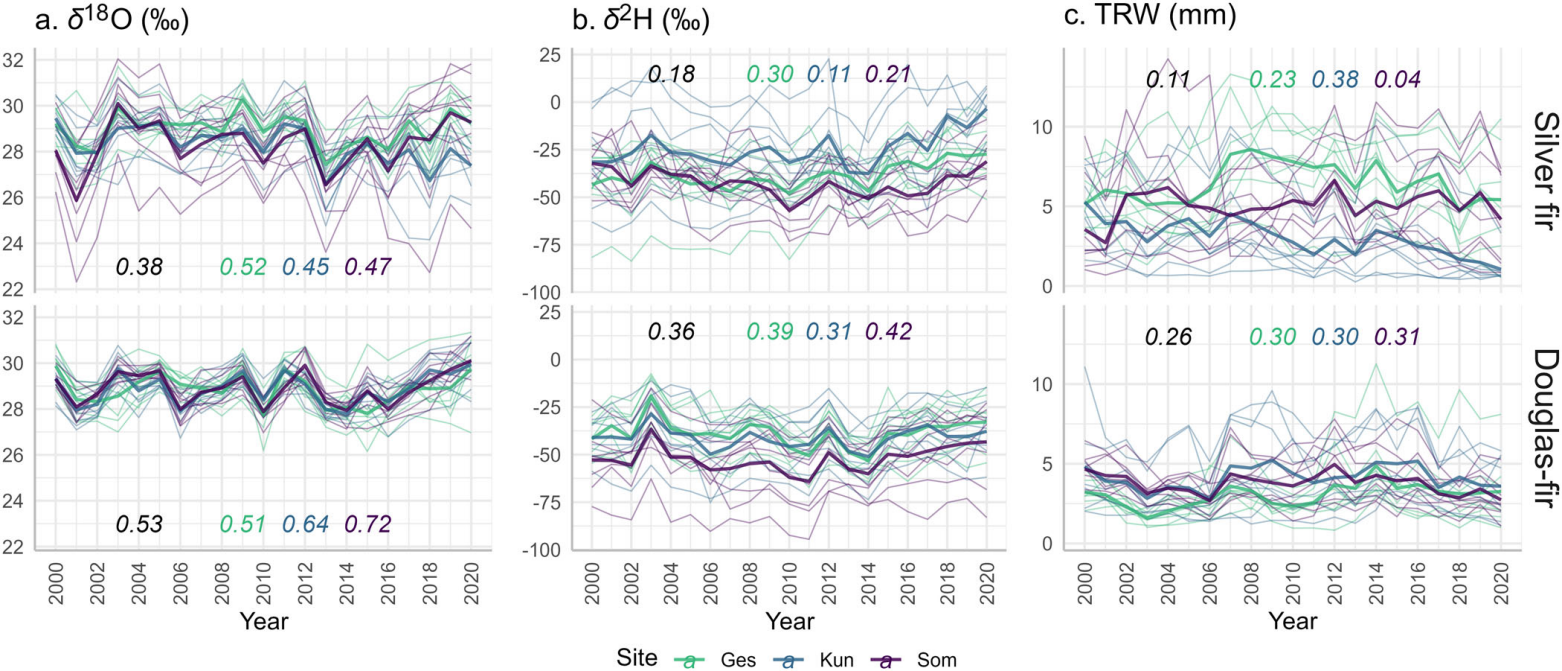
Tree individual time series and resulting chronologies of (a) *δ* ^18^O, (b) *δ* ^2^H and (c) TRW of silver fir (top) and Douglas‐fir (bottom). The thin lines show the individual series, and the thick lines indicate the mean site chronologies. The mean inter‐series correlation values are indicated per site (numbers in colour) and for the three sites combined (numbers in black) (see also Table S2). [Color figure can be viewed at wileyonlinelibrary.com]

Site chronologies were calculated as the mean of the eight trees per species and per site for *δ* ^18^O, *δ* ^2^H and TRW. To assess the common signal shared by all sampled individual trees at each site, we calculated the mean Pearson's correlation coefficient among the tree‐ring series ($\overset{®}{r}$) and the expressed population signal (Wigley, Briffa, and Jones 1984) (Table S2) with the function *rwi.stats* from the R package dplR (v1.7.4; Bunn et al. 2022). Similarly, to assess the coherence of the individual time series among sites, we calculated mean inter‐series correlations per tree‐ring variable and species (Figure 2).

To analyse the *δ* ^18^O–*δ* ^2^H relationship at the tree individual level (Research Question 1), we used one linear mixed‐effects model per species with tree identity included as a main effect and in interaction with *δ* ^18^O (*n* = 504 = 8 trees × 3 sites × 21 years; for each model). A random slope and random intercept were included for site identity (Model S1). Mixed‐effects models were calculated with the function *lme* from the R package *nlme* (v3.1‐162; Pinheiro, Bates, and Team 2023). We also analysed the *δ* ^18^O–*δ* ^2^H relationship at the site level (Research Question 1) with one mixed‐effects model per species using the average isotope values of the eight trees per year, site and species (*n *= 63 = 3 sites × 21 years; for each model). We included a random intercept and random slope for site identity (Model S2). In the models, we analysed *δ* ^2^H (variable *y*) in response to *δ* ^18^O (variable *x*), but the opposite was also possible since neither variable should explain the other and we simply analysed their relationship.

To analyse the relationship between each of the two isotope ratios and tree‐ring width (Research Question 2), we used additive mixed models, using the function *gam* from the package *mgcv* (v1.8‐42; Wood 2017). We calculated one model per species and per isotope ratio (four models in total with *n *= 504 = 8 trees × 3 sites × 21 years; for each model) using thin‐plate splines and restricted minimum likelihood. We included the site effect as a grouping factor and tree identity as a random effect. To avoid overfitting the data, we applied a penalization of *k *= 4 (Model S3). We repeated the same analysis at the individual tree level (four models in total, *n *= 504 for each model), this time grouping the data per tree identity and including the site effect as a random effect (Model S4).

To test the effect of tree age on *δ* ^18^O and *δ* ^2^H (Research Question 2), we also fitted linear mixed‐effects models by analysing each site separately based on tree individual data. For this, we fitted one model per species, site and isotope ratio (12 models in total, *n *= 168 = 8 trees × 21 years; for each model). To account for potential tree individual offsets, we included a random intercept for tree identity (Model S5).

To analyse the relationship between isotope ratios and tree crown volume (Research Question 2), we fitted one linear mixed‐effects model per species and per isotope ratio (four models in total, *n *= 24 = 8 trees × 3 sites; for each model). As response variable, we used the average isotope ratio of each tree over the last 5 years (2016–2020). We chose to average the last 5 years of isotope data because we measured the crown volume only once in 2020 or 2021. To account for differences among sites, we included a random intercept and random slope for site identity (Model S6).

To assess the climate sensitivity of *δ* ^18^O, *δ* ^2^H and TRW (Research Question 3), we calculated Pearson's correlation coefficients with a moving window between site chronologies and daily average temperature, precipitation sum, daily average global radiation and VPD for each site and each species. We used a modified version of the function *daily_response* from the package *dendroTools* (v1.2.8; Jevšenak 2020) to plot the results.

To analyse the influence of climate on the *δ* ^18^O–*δ* ^2^H relationship (Research Question 3), we first calculated Pearson's correlation coefficients between *δ* ^18^O and *δ* ^2^H per species and year. We decided to pool the three sites together to increase the sample size (24 pairs of values, 8 per site) and have more robust correlation values. We then fitted a linear model for each species between the correlation values and the yearly average VPD over the months of July to September taking the mean of the three study sites (two models in total, *n* = 21 = 21 yearly correlation values; for each model, Model S7). We also tested the period April to June, and the average temperature or precipitation sum (10 models in total, *n *= 21 for each model; Model S8), but the VPD in summer had the strongest effect on the *δ* ^18^O–*δ* ^2^H correlation. For this reason, we present the results based on summer VPD as the main result and include the analyses based on average temperature and precipitation sum and on spring climatic conditions as given in the Supporting Material.

To consider the influence of the isotopic signature of precipitation on our tree‐ring cellulose isotope values, we calculated bootstrapped Pearson's correlation coefficients with the function *dcc* from the R package *treeclim* (v2.0.6.0; Zang and Biondi 2015) between our annual tree‐ring isotope ratios and monthly modelled data of isotope ratios from precipitation (Figure S16).

All statistical analyses were conducted with the software R (v 4.2.2; R Core Team 2022) and the figures were plotted with *ggplot2* (v3.4.2; Wickham 2016), except Figure 1, made with ArcGIS Desktop v10.8. We tested for autocorrelation in our response variables (*δ* ^18^O and *δ* ^2^H) and did not include an autocorrelation parameter in our models because it was not prominent in our data. All models were assessed with diagnostic plots to check the normality of the distribution of errors and random effects.

## 3 Results

### 3.1 Time Series of the Tree‐Ring Variables

The *δ* ^18^O time series of the eight trees within a site were strongly correlated, and this was true at all sites and for both species, with slightly higher correlations for Douglas‐fir than for silver fir (Figure 2a). Further, the individual time series were also correlated among sites, with higher correlations for Douglas‐fir (0.53) than silver fir (0.38). Interestingly, *δ* ^18^O of Douglas‐fir shared the same range of values and did not show an offset among sites (Figure 2a). In comparison, the *δ* ^2^H time series of the individual trees were less correlated within sites than the *δ* ^18^O time series but we still observed positive correlations among individual series, especially for Douglas‐fir (Figure 2b). Among sites, *δ* ^2^H individual time series were also more strongly correlated for Douglas‐fir (0.36) than silver fir (0.18). Finally, TRW individual time series of both species were even less correlated within sites than *δ* ^18^O and *δ* ^2^H (Figure 2c). Among sites, Douglas‐fir individual series were also more strongly correlated (0.26) than silver fir (0.11).

### 3.2 *δ* ^18^O–*δ* ^2^H Relationship in Tree‐Ring Cellulose

**Figure 3:**
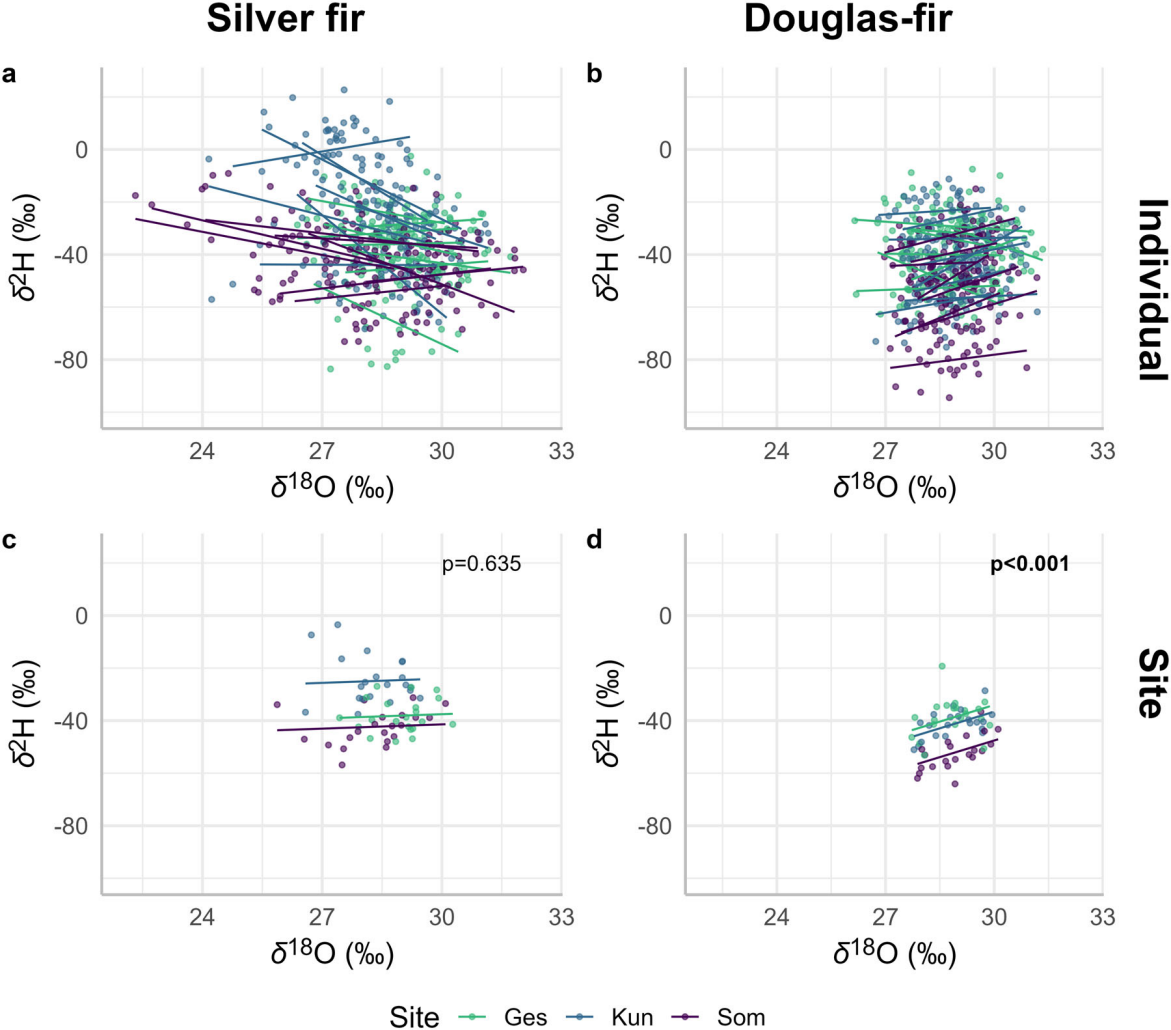
*δ* ^18^O–*δ* ^2^H relationship in tree‐ring cellulose (a, b) at the tree individual level for silver fir and Douglas‐fir, respectively. The dots show the raw data for the period 2000–2020, and the lines represent the fitted values for each tree following Model S1. The summaries of the models are presented in Table S3. (c, d) The *δ* ^18^O–*δ* ^2^H relationship at the site level is shown for silver fir and Douglas‐fir, respectively. The dots represent the raw data of the site chronologies (mean of *δ* ^18^O and *δ* ^2^H values of eight trees per year and per site), and the lines represent the fitted values for each site following Model S2. The *p*‐values correspond to the overall effect. The summaries of the models are presented in Table S4. [Color figure can be viewed at wileyonlinelibrary.com]

At the individual tree level, the overall *δ* ^18^O–*δ* ^2^H relationship was not significant for silver fir and we observed a high variability among trees, with significant interactions between tree identity and *δ* ^18^O for four trees (Figure 3a and Table S3). For Douglas‐fir, we observed an overall significant negative *δ* ^18^O–*δ* ^2^H relationship. However, the individual tree identity had strong and significant interactions with *δ* ^18^O for 16 trees (Table S3), which changed the strength and direction of the *δ* ^18^O–*δ* ^2^H relationship depending on the tree (Figure 3b). Therefore, we observed a high variability in the *δ* ^18^O–*δ* ^2^H relationship at the individual tree level. At the site level, the relationship remained non‐significant for silver fir (Figure 3c) but became significantly positive for Douglas‐fir (Figure 3d and Table S4).

### 3.3 Relationships With TRW, Tree Age and Crown Volume

**Figure 4:**
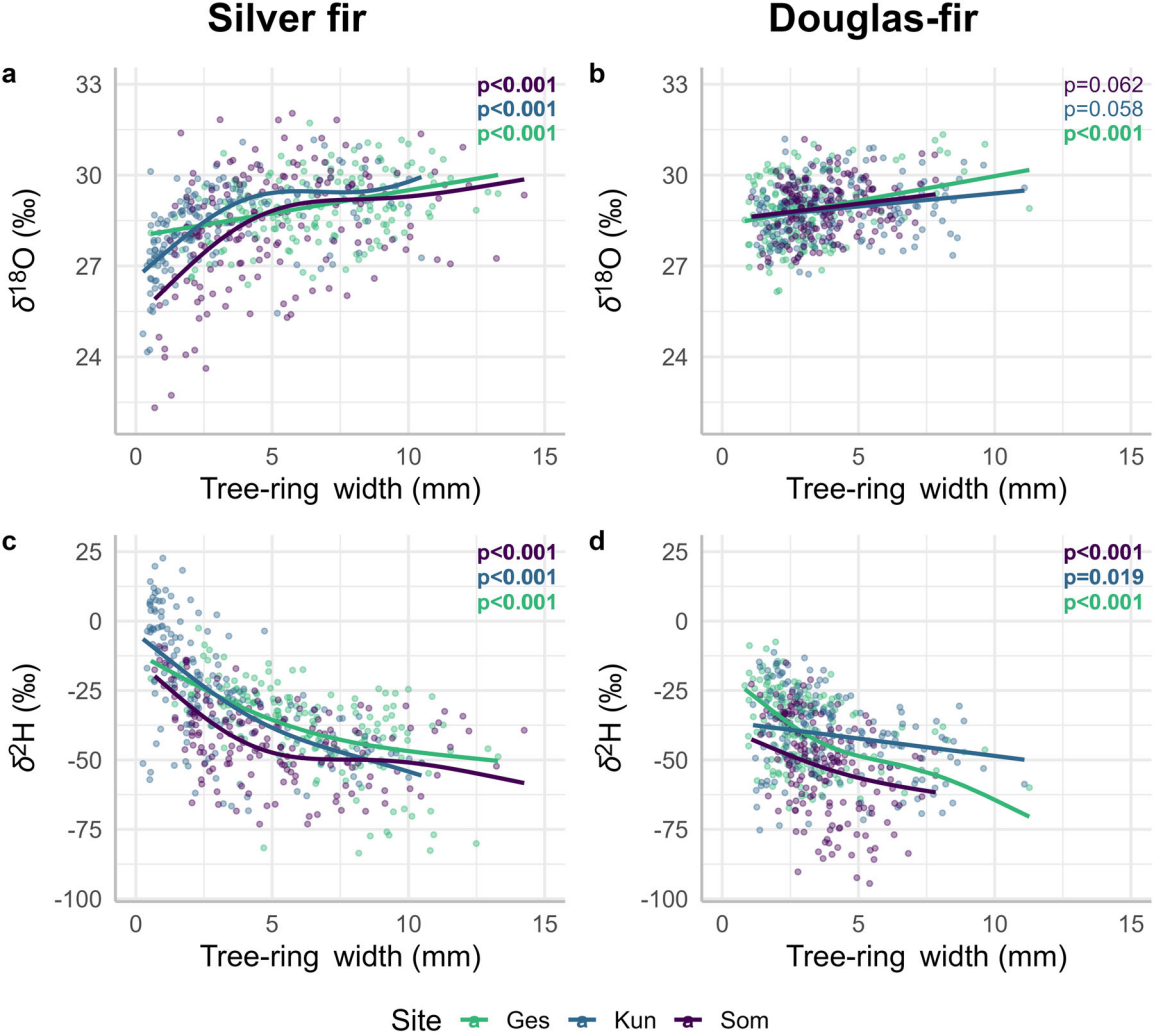
Relationship between *δ* ^18^O and TRW for (a) silver fir and (b) Douglas‐fir, and between *δ* ^2^H and TRW for (c) silver fir and (d) Douglas‐fir. The points show the raw data of the individual trees per site (see legend). The curves are fitted with additive mixed models following Model S3. The *p*‐values for each site are indicated in the top right corner of each subplot, with significant *p*‐values (≤ 0.05) in bold. The summaries of the model are presented in Table S5. [Color figure can be viewed at wileyonlinelibrary.com]

At all three sites, *δ* ^18^O and TRW of silver fir showed a significant positive relationship (Figure 4a and Table S5). For Douglas‐fir, this relationship was only significant at one site (Ges, Figure 4b). Between *δ* ^2^H and TRW, we found a significant, negative and non‐linear relationship at all sites and for both species, i.e., narrow tree rings were more enriched in ^2^H than wide tree rings (Figure 4c–d). At the individual tree level, we also observed a positive relationship between *δ* ^18^O and TRW, which was significant for most of the silver fir trees (Figure S2) and for fewer Douglas‐fir trees (Figure S3). The relationship between *δ* ^2^H and TRW was significantly negative for most of the silver fir trees (Figure S4) but weaker for Douglas‐fir trees (Figure S5).

**Figure 5:**
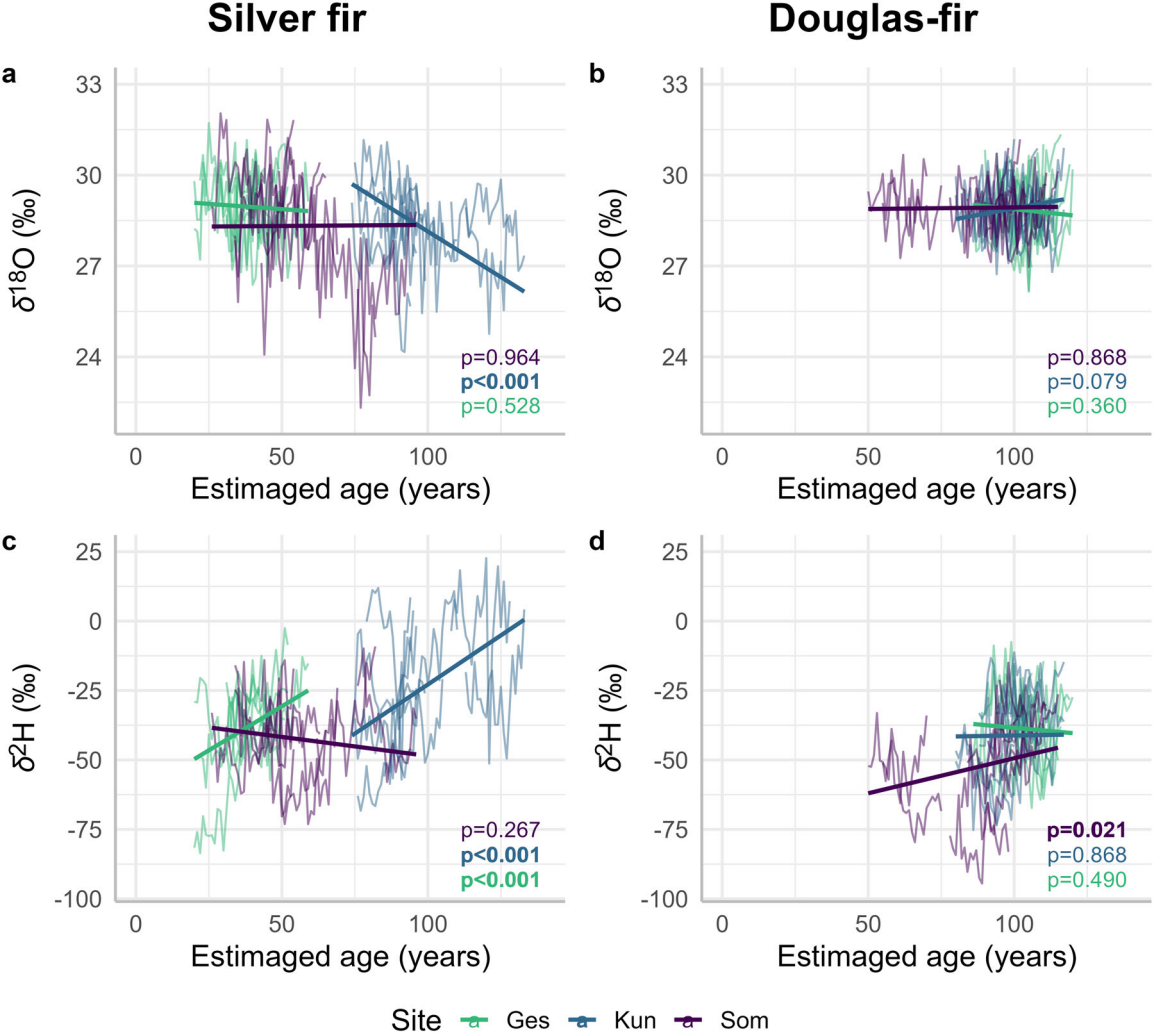
Relationships between tree age and tree‐ring *δ* ^18^O for (a) silver fir and (b) Douglas‐fir, and *δ* ^2^H of (c) silver fir and (d) Douglas‐fir. The fitted relationships are indicated by the thick coloured lines corresponding to the site. Each coloured time series represents an individual tree (21 years of data per tree). One mixed‐effects model was fitted per site, species and isotope ratio following Model S5. The outputs of the models are shown in Table S6. The *p*‐values are shown on each panel and for each site, with significant *p*‐values (≤ 0.05) in bold. [Color figure can be viewed at wileyonlinelibrary.com]

**Figure 6:**
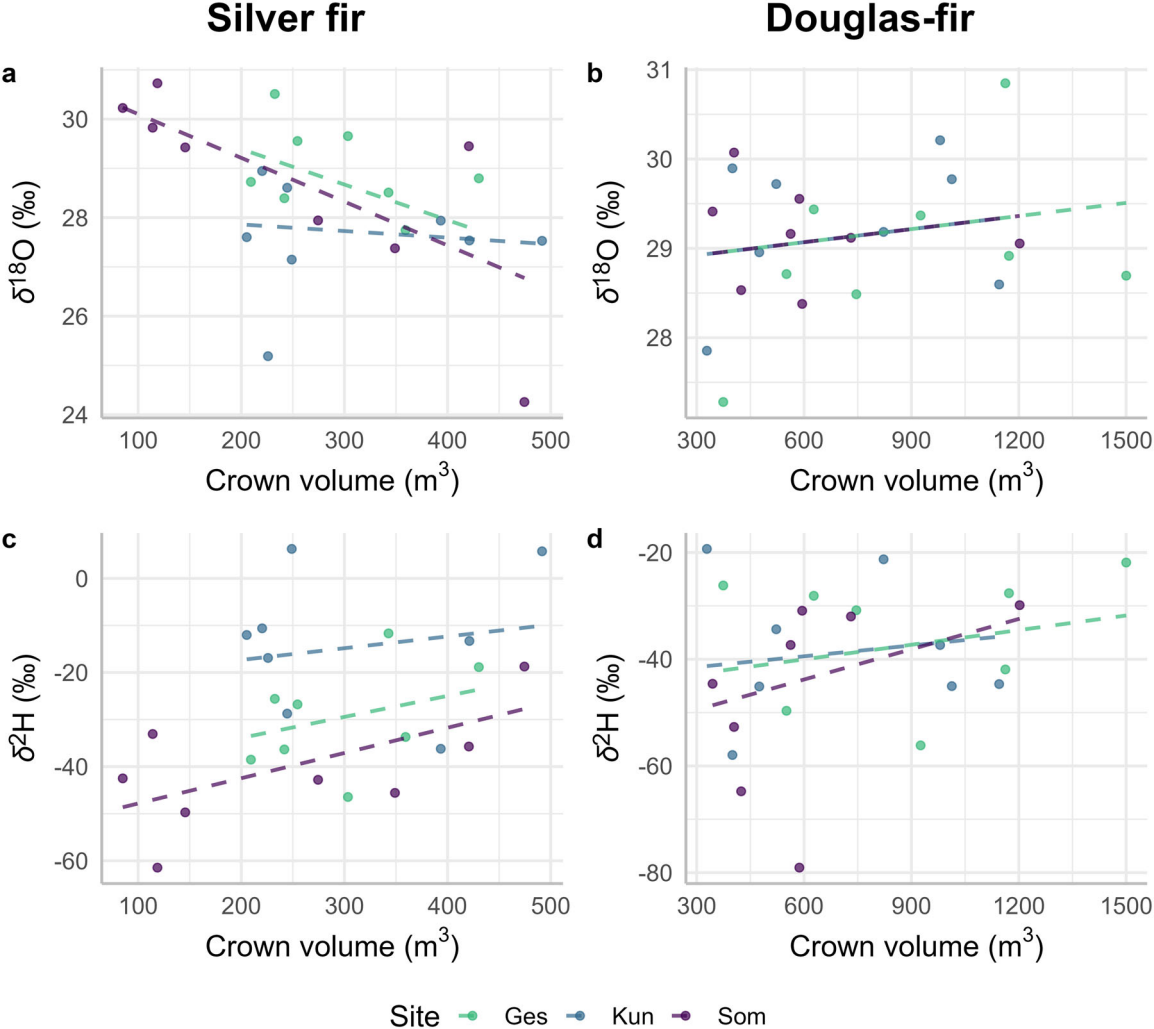
Relationships between crown volume and tree‐ring *δ* ^18^O of (a) silver fir and (b) Douglas‐fir, and *δ* ^2^H of (c) silver fir and (d) Douglas‐fir. The crown volume was measured in 2020 and each dot represents the average isotope ratio over the last five years (2016–2020). One mixed‐effects model was fitted per species and per isotope ratio following Model S6. The dashed lines represent the fitted relationships (crown volume had a non‐significant effect in all cases). The outputs of the models are shown in Table S7. [Color figure can be viewed at wileyonlinelibrary.com]

The relationship between *δ* ^18^O and tree age was significantly negative at the site Kun for silver fir (Figure 5a). Between *δ* ^2^H and tree age, we found a significant positive relationship at the sites Ges and Kun for silver fir (Figure 5c). Across sites, we could observe for silver fir a negative age trend in *δ* ^18^O and a positive age trend in *δ* ^2^H, although we did not test it statistically (see Discussion). For Douglas‐fir, the relationship between *δ* ^18^O and tree age was not significant (Figure 5b), and between *δ* ^2^H and tree age, it was significantly positive at the site Som only (Figure 5d and Table S6). Tree crown volume and tree‐ring *δ* ^18^O and *δ* ^2^H were not significantly related neither for silver fir nor for Douglas‐fir (Figure 6 and Table S7). Albeit not significant, for silver fir, *δ* ^18^O was negatively related to crown volume, whereas *δ* ^2^H was positively related to crown volume.

### 3.4 Climate Sensitivity of *δ* ^18^O and *δ* ^2^H

**Figure 7:**
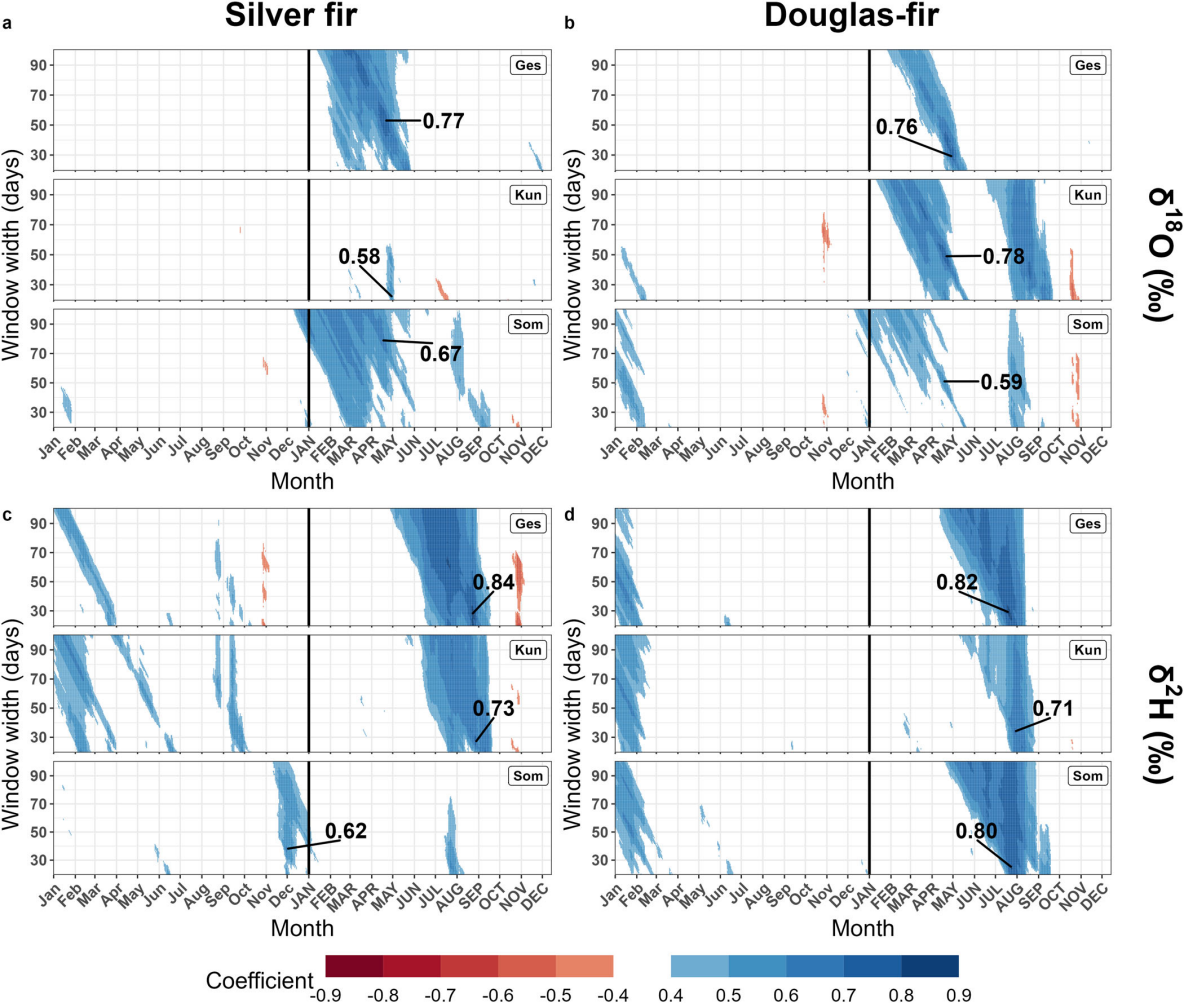
Moving‐window correlations between daily vapour pressure deficit (VPD) and tree‐ring chronologies of (a) *δ* ^18^O of silver fir, (b) *δ* ^18^O of Douglas‐fir, (c) *δ* ^2^H of silver fir and (d) *δ* ^2^H of Douglas‐fir for the period 2000–2020 at the three study sites (labels on the top right corners). Only significant correlations are shown (*p*‐value ≤ 0.05). The date on the *x*‐axis corresponds to the beginning of the window width (e.g., a point at 60 days on the *y*‐axis and the beginning of July on the *x*‐axis corresponds to a correlation with the climate data of 60 days starting at the beginning of July, i.e., covering July and August). Months in lowercase denote the months of the year before tree‐ring formation. Numbers in bold indicate the highest correlation per site. [Color figure can be viewed at wileyonlinelibrary.com]

Our analysis with daily climate data and moving windows of variable lengths showed that *δ* ^18^O was strongly positively correlated with VPD in late winter and spring for silver fir at two sites (Ges and Som) and for Douglas‐fir at all three sites (Figure 7a–b). In addition, *δ* ^18^O of Douglas‐fir was positively correlated with late summer VPD at the site Kun and, to a lesser extent, at the site Som (Figure 7b). For both species, the highest correlations were observed for window lengths between 25 and 80 days, starting around mid‐April (Figure 7a–b). Further, *δ* ^18^O correlated significantly and negatively with the precipitation sum at the end of winter/spring, except for silver fir at the site Kun and Douglas‐fir at the site Ges (Figure S6). Correlations between *δ* ^18^O and global radiation were positive and significant predominantly at the end of winter/spring, with stronger correlations for silver fir than Douglas‐fir (Figure S8).

Overall, *δ* ^2^H showed a strong and consistent positive relationship with summer VPD for silver fir at two sites (Ges and Kun) and Douglas‐fir at all three sites (Figure 7c,d). The highest correlations were observed for a window length of around 30 days, starting in mid‐August for silver fir and mid‐July for Douglas‐fir (Figure 7c,d). Further, *δ* ^2^H showed strong positive correlations to summer/late summer temperatures, particularly with a window length of around 60 days from mid‐July (Figure S7). We also observed positive correlations between *δ* ^2^H and global radiation in the summer months, with stronger correlations for silver fir than for Douglas‐fir, except at the site Som (Figure S8). Based on the strength, significance and time periods of the correlations, we inferred the climate sensitivity of *δ* ^18^O and *δ* ^2^H in silver fir and Douglas‐fir.

**Figure 8:**
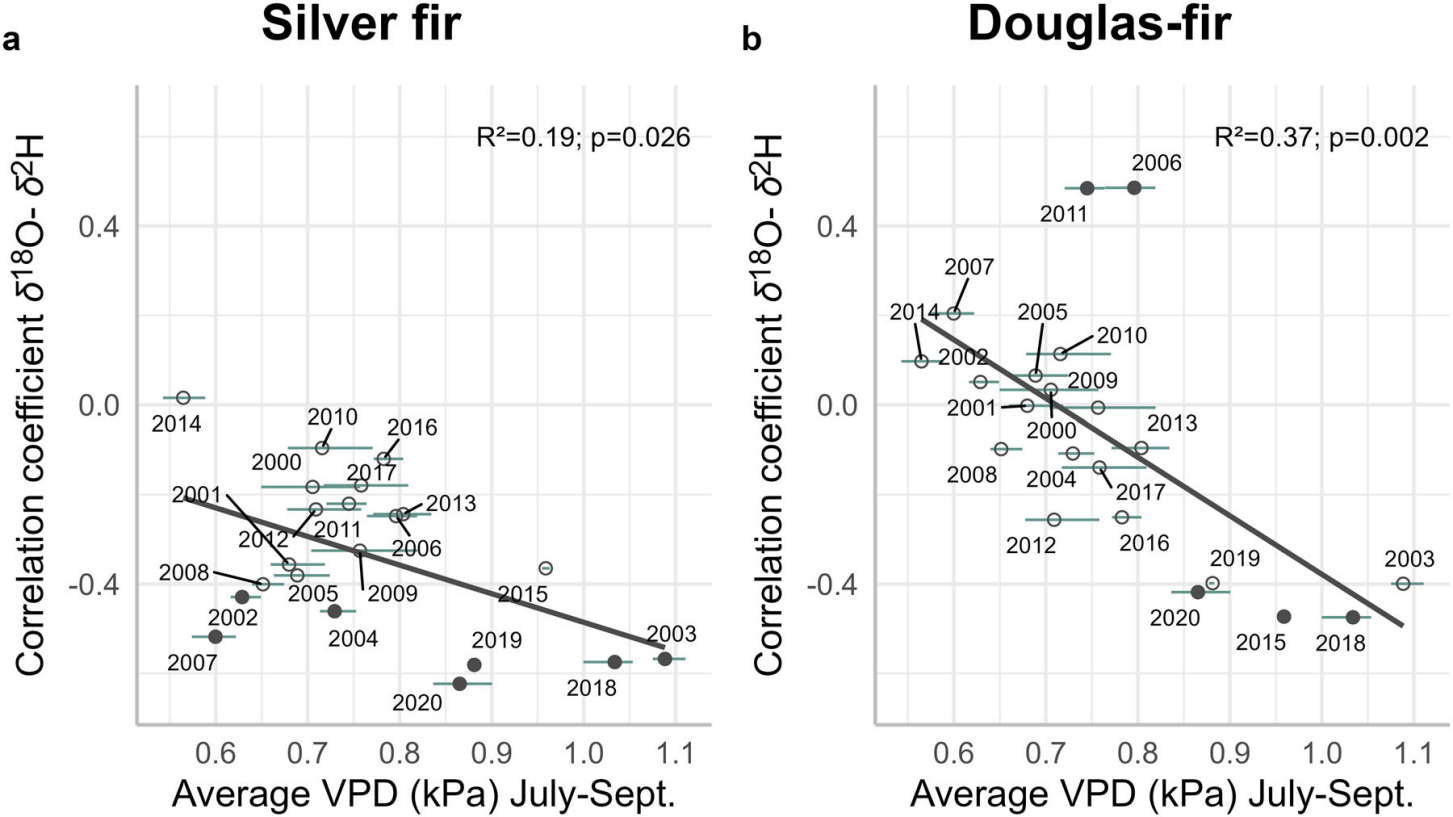
Relationship between the *δ* ^18^O–*δ* ^2^H correlation coefficients and the average summer vapour pressure deficit (VPD; July–September) for (a) silver fir and (b) Douglas‐fir. The filled and empty circles represent significant and non‐significant values of Pearson's correlation coefficients (*p* ≤ 0.05), respectively. Each correlation coefficient includes 24 pairs of *δ* ^18^O–*δ* ^2^H values (three sites pooled with eight trees each). The dark solid line on each panel shows the fitted relationships following Model S7. The horizontal lines overlapping each circle show the range of VPD values for the three sites for a given year. Adjusted *R* ^2^ and *p*‐values are shown for each fitted model. The outputs of the models are given in Table S8. [Color figure can be viewed at wileyonlinelibrary.com]

Despite the generally weak to absent correlation between *δ* ^18^O and *δ* ^2^H in tree‐ring cellulose, we nevertheless tested if the strength of their correlation coefficients was related to climatic conditions. Interestingly, we found stronger and negative *δ* ^18^O–*δ* ^2^H correlation coefficients under higher VPD conditions (Figure 8 and Table S8). The relationship between *δ* ^18^O–*δ* ^2^H correlation coefficients and summer VPD was negative for both species and was even more pronounced for Douglas‐fir (silver fir: *R* ^2^ = 0.19, *p* = 0.026; Douglas‐fir: *R* ^2^ = 0.37, *p*= 0.002; Figure 8). Specifically, during dry summers (e.g., 2018), the *δ* ^18^O–*δ* ^2^H relationship was highly negative for both species (Figure 8). We found, however, also exceptions, such as in 2006 and 2011 for Douglas‐fir, with relatively high positive *δ* ^18^O–*δ* ^2^H correlations despite medium summer VPD values (Figure 8b). The detailed *δ* ^18^O–*δ* ^2^H relationships for each year and species are presented in Figure S9. Summer VPD (July–September) had the strongest influence for both species on the *δ* ^18^O–*δ* ^2^H relationship. For Douglas‐fir, summer average temperature and precipitation sum had also significant negative and positive effects, respectively, on the *δ* ^18^O–*δ* ^2^H relationship (Figures S10 and S11). Spring climatic conditions (April–June) had no effect on the *δ* ^18^O–*δ* ^2^H relationship for both species when considering VPD, average temperature or precipitation sum (Figures S12–S14).

## 4 Discussion

### 4.1 *δ* ^18^O–*δ* ^2^H Relationship in Tree‐Ring Cellulose

The *δ* ^18^O–*δ* ^2^H relationship at the individual tree level was highly variable from tree to tree, and there was no clear tendency. This decoupling confirms observations from previous studies that also found a weak or absent correlation between *δ* ^18^O and *δ* ^2^H in tree‐ring cellulose (e.g., Holloway‐Phillips et al. 2023; Nabeshima et al. 2018 [in earlywood]; Vitali et al. 2022, 2024). At the site level, i.e., when combining eight individuals, the *δ* ^18^O–*δ* ^2^H relationship was still non‐significant for silver fir, but slightly positive for Douglas‐fir (Figure 3c–d). Although *δ* ^18^O and *δ* ^2^H originate from meteoric water (cf. Introduction) and we observed an almost perfectly linear relationship between *δ* ^18^O and *δ* ^2^H in precipitation at our sites (Figure S15c), nevertheless the weak *δ* ^18^O–*δ* ^2^H correlations in tree‐ring cellulose suggest that *δ* ^18^O and *δ* ^2^H are undergoing different isotope fractionation pathways after photosynthesis. This *δ* ^18^O–*δ* ^2^H decoupling seems to also occur at the level of leaf cellulose and to a greater extent in branch wood cellulose (Holloway‐Phillips et al. 2023). Nabeshima et al. (2018) found in *Quercus crispula* (Blume) in Japan a clear positive *δ* ^18^O–*δ* ^2^H relationship in latewood but not in earlywood. With intra‐annual measurements, the authors observed that *δ* ^2^H decreased through the year while *δ* ^18^O first increased and then decreased later in the growing season, explaining the correlation in latewood but not in earlywood. However, the intra‐annual *δ* ^2^H pattern is not clearly known and seems to vary across species and studies, although it seems that the beginning of the tree ring is often ^2^H‐enriched compared to the rest of the ring (Lehmann et al. 2022). In our study, we analysed the whole ring because there was a gradual change from early‐ to latewood, which hinders a clear separation but we acknowledge that the separate analysis of early‐ and latewood could provide additional information. In the following sections, we discuss potential explanations for the observed decoupling between *δ* ^18^O and *δ* ^2^H in tree‐ring cellulose.

### 4.2 Relationships With TRW, Tree Age and Crown Volume

We observed an overall positive relationship between *δ* ^18^O and TRW for both species (Figure 4a,b). For silver fir, the relationship was non‐linear and was especially strong for narrow tree rings (< 5 mm), while it lost strength for wide tree rings (> 5 mm). The positive relationship is surprising, given that smaller TRW and higher *δ* ^18^O are usually associated with dry climatic conditions in temperate regions (Andreu‐Hayles et al. 2017 and see Figure S17 for the correlations between TRW and VPD). We would have, therefore, expected a negative relationship between *δ* ^18^O and TRW. However, the negative correlations between TRW and VPD were mostly observed in late spring/summer, whereas the positive correlations between *δ* ^18^O and VPD were mostly observed in late winter/spring (see Section 4.3). This could explain the absence of a negative relationship between *δ* ^18^O and TRW, but not necessarily the positive relationship. The reason for the positive relationship between *δ* ^18^O and TRW could be that, in association with dry periods, trees access water from deeper, ^18^O‐depleted soil layers (Sarris, Siegwolf, and Körner 2013). However, deeper soil water is also ^2^H‐depleted, but the opposite relationship was observed for TRW‐*δ* ^2^H (see thereafter). Similar to our results, Lévesque et al. (2013) found a positive correlation between latewood width and latewood *δ* ^18^O in Douglas‐fir, with stronger correlations at a xeric than at a mesic site, the latter located in the same region as our sites and with similar climatic conditions. For silver fir, the positive relationship between *δ* ^18^O and TRW was also observed at the individual level (Figure S2). This indicates that the observed pattern is likely not due to age because trees had different age ranges across sites but still showed the same pattern, and each tree had only 21 years of data. For Douglas‐fir, the weaker relationship could be partly explained by the fewer data covering narrow tree rings, where we found the strongest relationship in silver fir (non‐linear relationship).

In striking contrast to the relationship with *δ* ^18^O, we found a clear negative *δ* ^2^H–TRW relationship for both species at all three sites (Figure 4c,d). As for TRW‐*δ* ^18^O, the *δ* ^2^H–TRW relationship was nonlinear, indicating a stronger link between TRW and *δ* ^2^H for narrow tree rings. This negative *δ* ^2^H–TRW relationship was expected, assuming that climate is the main driver of interannual variations because unfavourable climatic conditions such as drought usually cause an increase in *δ* ^2^H (see climate correlations in Figure 7c,d, Figures S6c,d and S7c,d) and a decrease in TRW (Fritts 1976, and see Figure S17). According to Lehmann et al. (2021), *δ* ^2^H reflects the use of stored carbohydrates, which are hypothesized to be ^2^H‐enriched. Therefore, *δ* ^2^H should be higher under more stressful conditions due to the increased use of stored carbohydrates, which would indeed match our observed high *δ* ^2^H values associated with low TRW (Figure 4c,d). In our study, the clear negative *δ* ^2^H–TRW relationship, even at the individual level and for both species, although less pronounced for Douglas‐fir (Figures S4 and S5), shows that some common factors partly drive *δ* ^2^H and TRW. In summary, we observed clear and contrasting relationships to TRW when looking at *δ* ^18^O or *δ* ^2^H. These observations are evidence for the decoupling between *δ* ^18^O and *δ* ^2^H in tree‐ring cellulose since *δ* ^18^O and *δ* ^2^H must have a different isotope fractionation pathway, leading to such opposite relationships to TRW. Further, the difference between *δ* ^18^O or *δ* ^2^H in correlation to TRW can suggest that early‐season growth conditions do not necessarily reflect late‐season growth conditions.

Whether or not tree‐ring stable isotope series present an age trend is still under debate in the literature, and this depends on the isotope ratio, species, site conditions, and age range analysed (Frank, Fang, and Fonti 2022). Some studies showed evidence for an absence of age trend over long time scales, for example, Büntgen et al. (2020) for *δ* ^18^O in oaks in Central Europe and Torbenson et al. (2022) for *δ* ^18^O in Scots pine (*Pinus sylvestris* L.) in boreal forests. In contrast, some other studies found an age trend. For example, Klesse et al. (2018) found mostly positive age trends for *δ* ^18^O in Norway spruce and European beech in a temperate forest and Mayr et al. (2003) observed a positive age trend for *δ* ^2^H in oaks in southern Germany. We observed that *δ* ^18^O significantly decreased with age at one site for silver fir. Across sites, i.e., when combining trees from all sites providing a larger age range, there was a general tendency for *δ* ^18^O to decrease with age (Figure 5a). Since deeper soil water pools are more ^18^O‐depleted (Treydte et al. 2014) than shallower ones due to soil surface evaporation, we would indeed expect older trees, which can develop longer roots over time, to access deeper soil water sources than younger trees, which is consistent, for example, with the results of Esper et al. (2010). We did not observe an effect of age on *δ* ^18^O for Douglas‐fir and the *δ* ^18^O values were stable through cambial age and across sites. As Arosio et al. (2020) described, the difference between the two species can be due to species‐specific response, with Douglas‐fir having rather a heart root system and silver fir a taproot. Regarding *δ* ^2^H, we observed that the values increased significantly with age at two sites for silver fir and one site for Douglas‐fir. In addition, we also observed a positive age trend in *δ* ^2^H across the three sites for silver fir. This observation does not support the rooting depth theory previously mentioned because shallower soils are ^2^H‐enriched (Dawson et al. 2002), or suggests that other factors are more important for *δ* ^2^H, overriding the source water effect. This positive age trend in tree‐ring *δ* ^2^H, described as the maturation effect in Lehmann et al. (2022), might be associated with the possibility to store and use carbohydrates or increased transport distance due to increased tree height. We did not statistically analyse the age effect across sites because of the potential isotope ratio variations in precipitation with geographical distances (although see the modelled data of isotopes in precipitation in Figure S15a‐b). Here, we hypothesize on the presence of age trends in *δ* ^18^O and *δ* ^2^H based on our observations, but we also acknowledge the limitation of our approach. However, analysing the age effect based on many short time series from trees of different ages growing at the same time instead of a single long time series avoids the long‐term effects of climate change. The bias introduced by individual tree variations should be offset by a large number of samples. Based on these observations, the contrasting age effect on *δ* ^18^O and *δ* ^2^H in tree‐ring cellulose, also observed by Nakatsuka et al. (2020), could be another evidence for their decoupling.

We could expect tree crown volume to be related to the isotope ratios because trees with a large crown have a large transpiring leaf area, affecting leaf water enrichment, but we did not observe this relationship. Such trees are also likely more vital, leading them to higher assimilation rates. Changes in photosynthetic assimilation rates and leaf water enrichment influence the isotope fractionation (McCarroll and Loader 2004). Further, it was shown that *δ* ^2^H in carbohydrates is affected by concentrations of sugars and starch together with leaf gas exchange (Lehmann et al. 2024). Therefore, trees with a larger crown might have lower isotope values than trees with a smaller crown, although very few studies have focused on this topic. Indeed, we expected trees with a larger crown to transpire more (shown for tree size; Dawson 1996; Gutierrez Lopez et al. 2021) and that a depletion of the heavy isotope would be reflected in the isotope ratios. In addition, the leaf water enrichment effect can be attenuated by high transpiration rates, due to the so‐called Péclet effect, where the continuous resupply of relatively unenriched source water from the soil diminishes the level of leaf water enrichment (Farquhar and Lloyd 1993). Tree crown volume can also reflect tree vitality, which has been shown to modulate the responses of tree‐ring stable isotope ratios to a strong change in water supply in Scots pine (Vitali et al. 2024). The reason why we did not find any significant effect of tree crown volume on *δ* ^18^O or *δ* ^2^H could be that our measurements of tree crown volume are not representative of the whole tree leaf area and thus of transpiration demand. Despite the absence of significant effects, we could observe, especially in silver fir, the same opposite effects of crown volume as observed with TRW or age on *δ* ^18^O and *δ* ^2^H, although age and crown volume were not significantly related for a given species within a site (results not shown). Overall, *δ* ^18^O and *δ* ^2^H were related very differently to tree intrinsic variables, showing that these two isotope ratios must undergo different isotope fractionation pathways or timings, and must be driven by different external factors such as climate.

### 4.3 Climate Sensitivity of *δ* ^18^O and *δ* ^2^H

We found a clear difference in the seasonal window of the strongest climate correlations for *δ* ^18^O compared to *δ* ^2^H. While spring conditions particularly influenced tree‐ring cellulose *δ* ^18^O, *δ* ^2^H values were more strongly related to summer climate. In the following, we discuss the reasons for this finding and the consequences on the *δ* ^18^O–*δ* ^2^H relationship. We observed that *δ* ^18^O in tree‐ring cellulose of both investigated species carries a strong late winter and spring precipitation and VPD signal. *δ* ^18^O in tree‐ring cellulose is partly influenced by the *δ* ^18^O signal from the source water (Martínez‐Sancho et al. 2023; Song, Lorrey, and Barbour 2022). In European temperate forests, winter precipitation is important for soil water replenishment and accounts for an important fraction of the water taken up by trees later during the growing season (Brinkmann et al. 2018). In addition, we observed the strongest correlations between *δ* ^18^O from tree‐ring cellulose and *δ* ^18^O from precipitation in March and May of the current year (Figure S16; March was the strongest for the three sites for silver fir), which indicates the influence of the source water signal, stored in the soil, from an earlier period in the year.

Previous studies suggested that *δ* ^2^H in tree‐ring cellulose carries a weaker climatic signal than *δ* ^18^O or *δ* ^13^C (Boettger et al. 2014; Loader et al. 2008; Vitali et al. 2022). In contrast, we observed strong correlations of *δ* ^2^H with summer to late summer climatic conditions, especially with temperature, for silver fir and Douglas‐fir. Such high correlations late in the growing season suggest the occurrence of strong post‐photosynthetic isotope fractionations during biochemical reactions for *δ* ^2^H or the exchange of carbon‐bound hydrogen between the carbohydrates and the water in the tree (Augusti, Betson, and Schleucher 2006). These processes could be important until late in the growing season and could be temperature‐driven, leading to a ^2^H enrichment in case of higher temperatures in the late summer. Solar radiation is also an important driver of photosynthesis and tree growth at Central European latitudes (Kašpar, Krůček, and Král 2024), likely influencing *δ* ^2^H (Lehmann et al. 2021). We observed some strong correlations between global radiation and *δ* ^2^H, particularly in summer for silver fir (Figure S8). Intense solar radiation enhances vapour pressure deficit, which reduces tree growth and photosynthesis (Kašpar, Krůček, and Král 2024) and could be related to an increase in tree‐ring *δ* ^2^H, as reported here. However, radiation and temperature/VPD are correlated, especially in the summer months (Figures S18 and S19) so the signal observed in *δ* ^2^H can be mixed between these variables. Summer climatic conditions have previously been reported to be correlated with tree‐ring cellulose *δ* ^2^H, although with a weaker strength (Boettger et al. 2014; Vitali et al. 2022). We also observed the highest correlations between *δ* ^2^H in tree‐ring cellulose and *δ* ^2^H in precipitation in July, but also in the previous winter (Figure S16). This mixed signal indicates that the roots can probably access deeper water stored in the soil from previous seasons and fresh water from the summer precipitation in upper soil layers. Because we found different climatic seasons influencing *δ* ^18^O and *δ* ^2^H, but that O and H originate from the same water source, this could indicate temporal differences in the fractionation of both isotope ratios, also suggested by Vitali et al. (2022) for pine and oak species.

These different climate sensitivities of *δ* ^18^O and *δ* ^2^H might be responsible for the generally weak *δ* ^18^O–*δ* ^2^H relationship. During high summer VPD, i.e., under drier and more stressful conditions, we found that the *δ* ^18^O–*δ* ^2^H relationship was reversing from the positive *δ* ^18^O–*δ* ^2^H found in water pools, with stronger negative correlations as summer VPD increased. An increase in VPD can be associated with lower stomatal conductance and photosynthetic rate but also increased transpiration rate for a given stomatal conductance, which leads to water loss for the plants (Grossiord et al. 2020). The strengthening of the *δ* ^18^O–*δ* ^2^H relationship was not observed when considering the spring climatic conditions (Figures S12–S14), and summer VPD was the only variable showing a significant effect for both species. For Douglas‐fir, we also observed strong negative and positive effects of summer temperature and precipitation sum on the *δ* ^18^O–*δ* ^2^H relationship, respectively (Figures S10 and S11). Our results suggest that water limitation in summer, i.e., stressful period, strengthens the negative *δ* ^18^O–*δ* ^2^H relationship. Summer seems to be a decisive season in the isotope fractionation for silver fir and Douglas‐fir at our study sites. Yet, we observed that *δ* ^2^H was strongly correlated with summer climatic conditions, whereas *δ* ^18^O was more strongly correlated with spring conditions, so *δ* ^2^H would be the main driver in the tightening and reversal of the *δ* ^18^O–*δ* ^2^H relationship. Biochemical effects, such as the mobilization of ^2^H‐enriched stored carbohydrates during stressful periods, have been shown to affect *δ* ^2^H independently from the hydrological signal and, therefore, independently from *δ* ^18^O (Lehmann et al. 2021). It seems that *δ* ^18^O and *δ* ^2^H are mostly uncorrelated under mesic conditions at our sites, maybe because there is not a single dominant environmental driver influencing the *δ* ^18^O–*δ* ^2^H relationship but multiple confounding effects. This could explain why we did not observe a clear and consistent link between *δ* ^18^O and *δ* ^2^H at the individual level because we included all years and most of them were not extreme in terms of climate. For Douglas‐fir, we observed an unexpected positive *δ* ^18^O–*δ* ^2^H correlation in 2006 and 2011. These years were neither particularly dry nor wet, and they were clearly not following the relationship observed between the *δ* ^18^O–*δ* ^2^H correlation coefficient and summer VPD. This could be related to masting years for Douglas‐fir, which affects resource allocation and storage and could thus affect the isotope ratios in tree‐ring cellulose. However, this is only a hypothesis, and we do not have masting data from our sites to prove it (but see Meng et al. 2022).

## 5 Conclusions

In our data set of *δ* ^18^O and *δ* ^2^H from tree‐ring cellulose of silver fir and Douglas‐fir, we observed the often‐reported decoupling between the two isotope ratios. To understand the reasons for this decoupling, we analysed separately each isotope ratio against tree‐ring width, tree age, tree crown volume and climate variables. We found striking differences in the relationships when focusing on *δ* ^18^O or *δ* ^2^H. In relation to tree‐ring width, we found a mirrored relationship with *δ* ^18^O and *δ* ^2^H. Concerning climate correlations, we found that not the same climate variable matters but also not the same season for *δ* ^18^O and *δ* ^2^H. All these differences help to explain the decoupling between *δ* ^18^O and *δ* ^2^H observed in tree‐ring cellulose. Further investigations at the intra‐annual level would be valuable to understand the physiological steps leading to these temporal differences. In addition, we found some differences in the results between silver fir and Douglas‐fir, suggesting that species‐specific studies are needed. We encourage further analyses to disentangle the presence or absence of age trends in tree‐ring stable isotope time series. Ultimately, the combined analysis of *δ* ^18^O and *δ* ^2^H in tree‐ring cellulose is a promising tool for deciphering tree physiological responses to ongoing climate change (e.g., a negative *δ* ^18^O–*δ* ^2^H relationship could indicate stressful climatic conditions). A deeper knowledge about *δ* ^18^O, *δ* ^2^H, and their decoupling is needed to understand the drought response of trees and might even help to identify species or individuals with stronger drought tolerance for forestry purposes in the future.

## Supporting information

Supporting information.

Supporting information.

## Data Availability

The data that support the findings of this study are openly available in EnviDat at https://doi.org/10.16904/envidat.527. The data set used for the analyses is available at https://doi.org/10.16904/envidat.527 (Charlet de Sauvage et al. 2024).

## References

[pce15252-bib-0001] Allen, R. G. , L. S. Pereira , D. Raes , M. Smith , et al. 1998. Crop Evapotranspiration – Guidelines for Computing Crop Water Requirements – FAO Irrigation and Drainage Paper 56 (p. 300). FAO – Food and Agriculture Organization of the United Nations.

[pce15252-bib-0002] Andreu‐Hayles, L. , C. C. Ummenhofer , M. Barriendos , et al. 2017. “400 Years of Summer Hydroclimate From Stable Isotopes in Iberian Trees.” Climate Dynamics 49, no. 1–2: 143–161. 10.1007/s00382-016-3332-z.

[pce15252-bib-0003] Arosio, T. , M. M. Ziehmer , K. Nicolussi , C. Schlüchter , and M. Leuenberger . 2020. “Alpine Holocene Tree‐Ring Dataset: Age‐Related Trends in the Stable Isotopes of Cellulose Show Species‐Specific Patterns.” Biogeosciences 17, no. 19: 4871–4882. 10.5194/bg-17-4871-2020.

[pce15252-bib-0004] Augusti, A. , T. R. Betson , and J. Schleucher . 2006. “Hydrogen Exchange During Cellulose Synthesis Distinguishes Climatic and Biochemical Isotope Fractionations in Tree Rings.” New Phytologist 172, no. 3: 490–499. 10.1111/j.1469-8137.2006.01843.x. 17083679

[pce15252-bib-0005] Augusti, A. , T. R. Betson , and J. Schleucher . 2008. “Deriving Correlated Climate and Physiological Signals From Deuterium Isotopomers in Tree Rings.” Chemical Geology 252, no. 1–2: 1–8. 10.1016/j.chemgeo.2008.01.011.

[pce15252-bib-0006] Boettger, T. , M. Haupt , M. Friedrich , and J. S. Waterhouse . 2014. “Reduced Climate Sensitivity of Carbon, Oxygen and Hydrogen Stable Isotope Ratios in Tree‐Ring Cellulose of Silver Fir (*Abies alba* Mill.) Influenced by Background SO_2_ in Franconia (Germany, Central Europe).” Environmental Pollution 185: 281–294. 10.1016/j.envpol.2013.10.030. 24316066

[pce15252-bib-0007] Boettger, T. , M. Haupt , K. Knöller , et al. 2007. “Wood Cellulose Preparation Methods and Mass Spectrometric Analyses of δ^13^C, δ^18^O, and Nonexchangeable δ^2^H Values in Cellulose, Sugar, and Starch: An Interlaboratory Comparison.” Analytical Chemistry 79, no. 12: 4603–4612. 10.1021/ac0700023. 17503767

[pce15252-bib-0008] Brinkmann, N. , S. Seeger , M. Weiler , N. Buchmann , W. Eugster , and A. Kahmen . 2018. “Employing Stable Isotopes to Determine the Residence Times of Soil Water and the Temporal Origin of Water Taken Up by *Fagus sylvatica* and *Picea abies* in a Temperate Forest.” New Phytologist 219, no. 4: 1300–1313. 10.1111/nph.15255. 29888480

[pce15252-bib-0009] Bunn, A. , M. Korpela , F. Biondi , et al. (2022). dplR: Dendrochronology Program Library in R. https://CRAN.R‐project.org/package=dplR.

[pce15252-bib-0010] Büntgen, U. , T. Kolář , M. Rybníček , et al. 2020. “No Age Trends in Oak Stable Isotopes.” Paleoceanography and Paleoclimatology 35, no. 4: e2019PA003831. 10.1029/2019PA003831.

[pce15252-bib-0011] Cernusak, L. A. , A. Barbeta , R. T. Bush , et al. 2022. “Do ^2^H and ^18^O in Leaf Water Reflect Environmental Drivers Differently?” New Phytologist 235, no. 1: 41–51. 10.1111/nph.18113. 35322882 PMC9322340

[pce15252-bib-0012] Cernusak, L. A. , M. M. Barbour , S. K. Arndt , et al. 2016. “Stable Isotopes in Leaf Water of Terrestrial Plants.” Plant, Cell & Environment 39, no. 5: 1087–1102. 10.1111/pce.12703. 26715126

[pce15252-bib-0013] Charlet de Sauvage, J. , H. Bugmann , C. Bigler , and M. Lévesque . 2023. “Species Diversity and Competition Have Minor Effects on the Growth Response of Silver Fir, European Larch and Douglas Fir to Drought.” Agricultural and Forest Meteorology 341: 109664. 10.1016/j.agrformet.2023.109664.

[pce15252-bib-0014] Charlet de Sauvage, J. , M. Saurer , K. Treydte , and M. Lévesque . 2024. *Carbon, Oxygen and Hydrogen Isotopes in Tree‐Ring Cellulose of Silver Fir and Douglas‐Fir in Switzerland* [Dataset]. EnviDat. 10.16904/envidat.527. PMC11247184 38874315

[pce15252-bib-0015] Craig, H. 1961. “Isotopic Variations in Meteoric Waters.” Science 133, no. 3465: 1702–1703. 10.1126/science.133.3465.1702. 17814749

[pce15252-bib-0016] Dawson, T. E. 1996. “Determining Water Use by Trees and Forests From Isotopic, Energy Balance and Transpiration Analyses: The Roles of Tree Size and Hydraulic Lift.” Tree Physiology 16, no. 1–2: 263–272. 10.1093/treephys/16.1-2.263. 14871771

[pce15252-bib-0017] Dawson, T. E. , S. Mambelli , A. H. Plamboeck , P. H. Templer , and K. P. Tu . 2002. “Stable Isotopes in Plant Ecology.” Annual Review of Ecology and Systematics 33, no. 1: 507–559. 10.1146/annurev.ecolsys.33.020602.095451.

[pce15252-bib-0018] DeNiro, M. J. , and S. Epstein . 1979. “Relationship Between the Oxygen Isotope Ratios of Terrestrial Plant Cellulose, Carbon Dioxide, and Water.” Science 204, no. 4388: 51–53. 10.1126/science.204.4388.51. 17816736

[pce15252-bib-0019] Dongmann, G. , H. W. Nürnberg , H. Förstel , and K. Wagener . 1974. “On the Enrichment of H_2_ ^18^O in the Leaves of Transpiring Plants.” Radiation and Environmental Biophysics 11, no. 1: 41–52. 4832051 10.1007/BF01323099

[pce15252-bib-0020] Esper, J. , D. C. Frank , G. Battipaglia , et al. 2010. “Low‐Frequency Noise in *δ* ^13^C and *δ* ^18^O Tree Ring Data: A Case Study of *Pinus uncinata* in the Spanish Pyrenees.” Global Biogeochemical Cycles 24, no. 4: 2010GB003772. 10.1029/2010GB003772.

[pce15252-bib-0021] Farquhar, G. D. , and J. Lloyd . 1993. “Carbon and Oxygen Isotope Effects in the Exchange of Carbon Dioxide Between Terrestrial Plants and the Atmosphere.” In Stable Isotopes and Plant Carbon‐Water Relations, edited by J. R. Ehleringer , A. E. Hall , and G. D. Farquhar , 47–70. San Diego: Elsevier. 10.1016/B978-0-08-091801-3.50011-8.

[pce15252-bib-0022] Frank, D. , K. Fang , and P. Fonti . 2022. “Dendrochronology: Fundamentals and Innovations.” In Stable Isotopes in Tree Rings: Inferring Physiological, Climatic and Environmental Responses, edited by R. T. W. Siegwolf , J. R. Brooks , J. Roden , and M. Saurer , 21–59. Cham: Springer International Publishing Cham.

[pce15252-bib-0023] Fritts, H. C. 1976. Tree Rings and Climate, edited by H. C. Fritts . London: Academic Press. 10.1016/B978-0-12-268450-0.X5001-0.

[pce15252-bib-0024] Grossiord, C. , T. N. Buckley , L. A. Cernusak , et al. 2020. “Plant Responses to Rising Vapor Pressure Deficit.” New Phytologist 226, no. 6: 1550–1566. 10.1111/nph.16485. 32064613

[pce15252-bib-0025] Gutierrez Lopez, J. , P. Tor‐ngern , R. Oren , N. Kozii , H. Laudon , and N. J. Hasselquist . 2021. “How Tree Species, Tree Size, and Topographical Location Influenced Tree Transpiration in Northern Boreal Forests during the Historic 2018 Drought.” Global Change Biology 27, no. 13: 3066–3078. 10.1111/gcb.15601. 33949757

[pce15252-bib-0026] Hartl‐Meier, C. , C. Zang , U. Buntgen , et al. 2015. “Uniform Climate Sensitivity in Tree‐Ring Stable Isotopes Across Species and Sites in a Mid‐Latitude Temperate Forest.” Tree Physiology 35, no. 1: 4–15. 10.1093/treephys/tpu096. 25466725

[pce15252-bib-0027] Holloway‐Phillips, M. , J. Baan , D. B. Nelson , M. M. Lehmann , G. Tcherkez , and A. Kahmen . 2022. “Species Variation in the Hydrogen Isotope Composition of Leaf Cellulose Is Mostly Driven by Isotopic Variation in Leaf Sucrose.” Plant, Cell & Environment 45, no. 9: 2636–2651. 10.1111/pce.14362 35609972

[pce15252-bib-0028] Holloway‐Phillips, M. , L. A. Cernusak , D. B. Nelson , M. M. Lehmann , G. Tcherkez , and A. Kahmen . 2023. “Covariation Between Oxygen and Hydrogen Stable Isotopes Declines Along the Path From Xylem Water to Wood Cellulose Across an Aridity Gradient.” New Phytologist 240: 1758–1773. 10.1111/nph.19248. 37680025

[pce15252-bib-0029] Holmes, R. L. 1983. “Computer‐Assisted Quality Control in Tree Ring Dating and Measurement.” Tree‐Ring Bulletin 43: 69–78.

[pce15252-bib-0030] Jevšenak, J. 2020. “New Features in the dendroTools R Package: Bootstrapped and Partial Correlation Coefficients for Monthly and Daily Climate Data.” Dendrochronologia 63: 125753. 10.1016/j.dendro.2020.125753.

[pce15252-bib-0031] Kašpar, J. , M. Krůček , and K. Král . 2024. “The Effects of Solar Radiation on Daily and Seasonal Stem Increment of Canopy Trees in European Temperate Old‐Growth Forests.” New Phytologist 243, no. 2: 662–673. 10.1111/nph.19852. 38769735

[pce15252-bib-0032] Klesse, S. , R. Weigt , K. Treydte , et al. 2018. “Oxygen Isotopes in Tree Rings Are Less Sensitive to Changes in Tree Size and Relative Canopy Position Than Carbon Isotopes.” Plant, Cell & Environment 41, no. 12: 2899–2914. 10.1111/pce.13424. 30107635

[pce15252-bib-0033] Laumer, W. , L. Andreu , G. Helle , G. H. Schleser , T. Wieloch , and H. Wissel . 2009. “A Novel Approach for the Homogenization of Cellulose to Use Micro‐Amounts for Stable Isotope Analyses.” Rapid Communications in Mass Spectrometry 23, no. 13: 1934–1940. 10.1002/rcm.4105. 19504486

[pce15252-bib-0034] Lehmann, M. M. , P. Schuler , M.‐A. Cormier , S. T. Allen , M. Leuenberger , and S. Voelker . 2022. “The Stable Hydrogen Isotopic Signature: From Source Water to Tree Rings.” In Stable Isotopes in Tree Rings: Inferring Physiological, Climatic and Environmental Responses, vol. 8, 331. Springer International Publishing. 10.1007/978-3-030-92698-4_11.

[pce15252-bib-0035] Lehmann, M. M. , P. Schuler , R. A. Werner , M. Saurer , G. L. B. Wiesenberg , and M.‐A. Cormier . 2024. “Biochemical and Biophysical Drivers of the Hydrogen Isotopic Composition of Carbohydrates and Acetogenic Lipids.” Science Advances 10, no. 28: eadl3591. 10.1126/sciadv.adl3591. 38985863 PMC11235168

[pce15252-bib-0036] Lehmann, M. M. , V. Vitali , P. Schuler , M. Leuenberger , and M. Saurer . 2021. “More Than Climate: Hydrogen Isotope Ratios in Tree Rings as Novel Plant Physiological Indicator for Stress Conditions.” Dendrochronologia 65: 125788. 10.1016/j.dendro.2020.125788.

[pce15252-bib-0037] Lévesque, M. , A. Rigling , H. Bugmann , P. Weber , and P. Brang . 2014. “Growth Response of Five Co‐Occurring Conifers to Drought Across a Wide Climatic Gradient in Central Europe.” Agricultural and Forest Meteorology 197: 1–12. 10.1016/j.agrformet.2014.06.001.

[pce15252-bib-0038] Lévesque, M. , M. Saurer , R. Siegwolf , et al. 2013. “Drought Response of Five Conifer Species Under Contrasting Water Availability Suggests High Vulnerability of Norway Spruce and European Larch.” Global Change Biology 19, no. 10: 3184–3199. 10.1111/gcb.12268. 23712589

[pce15252-bib-0039] Loader, N. J. , P. M. Santillo , J. P. Woodman‐Ralph , et al. 2008. “Multiple Stable Isotopes From Oak Trees in Southwestern Scotland and the Potential for Stable Isotope Dendroclimatology in Maritime Climatic Regions.” Chemical Geology 252, no. 1–2: 62–71. 10.1016/j.chemgeo.2008.01.006.

[pce15252-bib-0040] Lotter, A. F. , P. G. Appleby , R. Bindler , et al. 2002. “The Sediment Record of the Past 200 Years in a Swiss High‐Alpine Lake: Hagelseewli (2339 m a.s.l.).” Journal of Paleolimnology 28, no. 1: 111–127. 10.1023/A:1020328119961.

[pce15252-bib-0041] van der Maaten‐Theunissen, M. , H.‐P. Kahle , and E. van der Maaten . 2013. “Drought Sensitivity of Norway Spruce Is Higher Than That of Silver Fir Along an Altitudinal Gradient in Southwestern Germany.” Annals of Forest Science 70, no. 2: 185–193. 10.1007/s13595-012-0241-0.

[pce15252-bib-0042] Martínez‐Sancho, E. , L. A. Cernusak , P. Fonti , et al. 2023. “Unenriched Xylem Water Contribution During Cellulose Synthesis Influenced by Atmospheric Demand Governs the Intra‐Annual Tree‐Ring δ^18^O Signature.” New Phytologist 240: nph.19278. 10.1111/nph.19278. 37753542

[pce15252-bib-0043] Mayr, C. , B. Frenzel , M. Friedrich , M. Spurk , W. Stichler , and P. Trimborn . 2003. “Stable Carbon‐and Hydrogen‐Isotope Ratios of Subfossil Oaks in Southern Germany: Methodology and Application to a Composite Record for the Holocene.” Holocene 13, no. 3: 393–402.

[pce15252-bib-0044] McCarroll, D. , and N. J. Loader . 2004. “Stable Isotopes in Tree Rings.” Quaternary Science Reviews 23, no. 7–8: 771–801. 10.1016/j.quascirev.2003.06.017.

[pce15252-bib-0045] Meng, F. , Y. Yuan , S. Jung , B. Stimm , N. Estrella , and A. Menzel . 2022. “Long‐Term Flowering Intensity of European Tree Species Under the Influence of Climatic and Resource Dynamic Variables.” Agricultural and Forest Meteorology 323: 109074. 10.1016/j.agrformet.2022.109074.

[pce15252-bib-0046] Nabeshima, E. , T. Nakatsuka , A. Kagawa , T. Hiura , and R. Funada . 2018. “Seasonal Changes of δD and δ^18^O in Tree‐Ring Cellulose of *Quercus Crispula* Suggest a Change in Post‐Photosynthetic Processes During Earlywood Growth.” Tree Physiology 38, no. 12: 1829–1840. 10.1093/treephys/tpy068. 29920607

[pce15252-bib-0047] Nakatsuka, T. , M. Sano , Z. Li , et al. 2020. “A 2600‐year Summer Climate Reconstruction in Central Japan by Integrating Tree‐Ring Stable Oxygen and Hydrogen Isotopes.” Climate of the Past 16, no. 6: 2153–2172. 10.5194/cp-16-2153-2020.

[pce15252-bib-0048] Nelson, D. B. , D. Basler , and A. Kahmen (2021). “Precipitation Isotope Time Series Predictions From Machine Learning Applied in Europe.” Proceedings of the National Academy of Sciences, 118(26), e2024107118. 10.1073/pnas.2024107118. PMC8256050 34162705

[pce15252-bib-0049] Novick, K. A. , D. L. Ficklin , C. Grossiord , et al. 2024. “The Impacts of Rising Vapour Pressure Deficit in Natural and Managed Ecosystems.” Plant, Cell & Environment 47, no. 9: 3561–3589. 10.1111/pce.14846. 38348610

[pce15252-bib-0050] Pinheiro, J. , D. Bates , and R. Core Team . (2023). nlme: Linear and Nonlinear Mixed Effects Models. https://CRAN.R‐project.org/package=nlme.

[pce15252-bib-0051] R Core Team . (2022). R: A Language and Environment for Statistical Computing. R Foundation for Statistical Computing. https://www.R‐project.org/.

[pce15252-bib-0052] Roden, J. S. , G. Lin , and J. R. Ehleringer . 2000. “A Mechanistic Model for Interpretation of Hydrogen and Oxygen Isotope Ratios in Tree‐Ring Cellulose.” Geochimica et Cosmochimica Acta 64, no. 1: 21–35. 10.1016/S0016-7037(99)00195-7.

[pce15252-bib-0053] Sarris, D. , R. Siegwolf , and C. Körner . 2013. “Inter‐ and Intra‐Annual Stable Carbon and Oxygen Isotope Signals in Response to Drought in Mediterranean Pines.” Agricultural and Forest Meteorology 168: 59–68. 10.1016/j.agrformet.2012.08.007.

[pce15252-bib-0054] Saurer, M. , P. Cherubini , C. E. Reynolds‐Henne , K. S. Treydte , W. T. Anderson , and R. T. W. Siegwolf . 2008. “An Investigation of the Common Signal in Tree Ring Stable Isotope Chronologies at Temperate Sites.” Journal of Geophysical Research: Biogeosciences 113, no. G4: 2008JG000689. 10.1029/2008JG000689.

[pce15252-bib-0055] Schuler, P. , M. A. Cormier , R. A. Werner , et al. 2022. “A High‐Temperature Water Vapor Equilibration Method to Determine Non‐Exchangeable Hydrogen Isotope Ratios of Sugar, Starch and Cellulose.” Plant, Cell & Environment 45, no. 1: 12–22. 10.1111/pce.14193. PMC9291759 34564870

[pce15252-bib-0056] Song, X. , A. Lorrey , and M. M. Barbour . 2022. “Environmental, Physiological and Biochemical Processes Determining the Oxygen Isotope Ratio of Tree‐ring Cellulose.” In Stable Isotopes in Tree Rings: Inferring Physiological, Climatic and Environmental Responses, edited by R. T. W. Siegwolf , J. R. Brooks , J. Roden , and M. Saurer , 311–329. Cham: Springer International Publishing.

[pce15252-bib-0057] Spiecker, H. , M. Lindner , and J. K. Schuler , eds. 2019. Douglas‐Fir: An Option for Europe . Joensuu, Finland: European Forest Institute.

[pce15252-bib-0058] Sternberg, L. , M. C. Pinzon , W. T. Anderson , and A. H. Jahren . 2006. “Variation in Oxygen Isotope Fractionation During Cellulose Synthesis: Intramolecular and Biosynthetic Effects.” Plant, Cell & Environment 29, no. 10: 1881–1889. 10.1111/j.1365-3040.2006.01564.x. 16930314

[pce15252-bib-0059] Torbenson, M. , L. Klippel , C. Hartl , et al. 2022. “Investigation of Age Trends in Tree‐Ring Stable Carbon and Oxygen Isotopes From Northern Fennoscandia Over the Past Millennium.” Quaternary International 631: 105–114. 10.1016/j.quaint.2022.05.017.

[pce15252-bib-0060] Treydte, K. , S. Boda , E. Graf Pannatier , et al. 2014. “Seasonal Transfer of Oxygen Isotopes From Precipitation and Soil to the Tree Ring: Source Water Versus Needle Water Enrichment.” New Phytologist 202, no. 3: 772–783. 10.1111/nph.12741. 24602089

[pce15252-bib-0061] Treydte, K. , L. Liu , R. S. Padrón , et al. 2024. “Recent Human‐Induced Atmospheric Drying Across Europe Unprecedented in the Last 400 Years.” Nature Geoscience 17: 58–65.

[pce15252-bib-0062] Vitali, V. , U. Büntgen , and J. Bauhus . 2017. “Silver Fir and Douglas Fir Are More Tolerant to Extreme Droughts Than Norway Spruce in South‐Western Germany.” Global Change Biology 23, no. 12: 5108–5119. 10.1111/gcb.13774. 28556403

[pce15252-bib-0063] Vitali, V. , E. Martínez‐Sancho , K. Treydte , et al. 2022. “The Unknown Third – Hydrogen Isotopes in Tree‐Ring Cellulose Across Europe.” Science of The Total Environment 813: 152281. 10.1016/j.scitotenv.2021.152281. 34942249

[pce15252-bib-0064] Vitali, V. , R. L. Peters , M. M. Lehmann , et al. 2023. “Tree‐Ring Isotopes From the Swiss Alps Reveal Non‐Climatic Fingerprints of Cyclic Insect Population Outbreaks Over the Past 700 Years.” Tree Physiology 43, no. 5: 706–721. 10.1093/treephys/tpad014. 36738262 PMC10177004

[pce15252-bib-0065] Vitali, V. , P. Schuler , M. Holloway‐Phillips , et al. 2024. “Finding Balance: Tree‐Ring Isotopes Differentiate Between Acclimation and Stress‐Induced Imbalance in a Long‐Term Irrigation Experiment.” Global Change Biology 30, no. 3: e17237. 10.1111/gcb.17237. 38488024

[pce15252-bib-0066] Vitasse, Y. , A. Bottero , M. Rebetez , et al. 2019. “What Is the Potential of Silver Fir to Thrive Under Warmer and Drier Climate?” European Journal of Forest Research 138, no. 4: 547–560. 10.1007/s10342-019-01192-4.

[pce15252-bib-0067] Weigt, R. B. , S. Bräunlich , L. Zimmermann , et al. 2015. “Comparison of δ^18^O and δ^13^C Values Between Tree‐Ring Whole Wood and Cellulose in Five Species Growing Under Two Different Site Conditions.” Rapid Communications in Mass Spectrometry 29, no. 23: 2233–2244. 10.1002/rcm.7388. 26522315

[pce15252-bib-0068] Wickham, H. 2016. ggplot2: Elegant Graphics for Data Analysis. New York: Springer‐Verlag. https://ggplot2.tidyverse.org.

[pce15252-bib-0069] Wigley, T. M. L. , K. R. Briffa , and P. D. Jones . 1984. “On the Average Value of Correlated Time Series, With Applications in Dendroclimatology and Hydrometeorology.” Journal of Climate and Applied Meteorology 23, no. 2: 201–213.

[pce15252-bib-0070] Wolf, H. 2003. “Silver fir (*Abies alba*).” EUFORGEN Technical Guidelines for Genetic Conservation and Use.

[pce15252-bib-0071] Wood, S. N. 2017. Generalized Additive Models: An Introduction with R (2nd ed.). Chapman and Hall/CRC.

[pce15252-bib-0072] Zang, C. , and F. Biondi . 2015. “Treeclim: An R Package for the Numerical Calibration of Proxy‐Climate Relationships.” Ecography 38, no. 4: 431–436. 10.1111/ecog.01335.

