# Supplementary material for: Decoupling of Tree‐Ring Cellulose δ 18O and δ 2H Highlighted by Their Contrasting Relationships to Climate and Tree Intrinsic Variables
Source: Plant Cell Environ. 2024 Nov 7;48(3):1903–18. doi: 10.1111/pce.15252 (PMC11788974; doi:10.1111/pce.15252)
Supplement: Supplementary file 1 — Supporting information. [file PCE-48-1903-s002.pdf]

Decoupling of tree-ring cellulose δ^{18}O and δ^{2}H highlighted by their contrasting relationships to climate and tree intrinsic variables

Justine Charlet de Sauvage, Matthias Saurer, Kerstin Treydte, Mathieu Lévesque

Supplementary material

Supplementary Table 1. Description of the sites and of the meteorological stations used for the climate data. The full names of the sites are given together with the three-letter code used elsewhere in the text and figures. For latitude, longitude and aspect of the slope, north (N), south (S), west (W) and east (E) refer to the cardinal points. The mean annual temperature and annual precipitation sum are shown with mean ± standard deviation for the period 2000–2020, with the temperature adjusted for the difference in elevation between the site and the meteorological station.

	Bois des Gésiaux (Ges)	Küngoldingen (Kun)	Sommerwies (Som)
**Site description**			
Latitude (N)	46° 33' 20"	47° 18' 6"	47° 43' 6"
Longitude (E)	6° 39' 12"	7° 56' 50"	8° 36' 41"
Elevation (m a.s.l.)	760	480	550
Aspect	S	W	NE
Slope (°)	5	8	10
Temperature (°C)	9.9 ± 6.4	9.8 ± 6.7	9.5 ± 6.8
Precipitation (mm)	1094 ± 219	942 ± 144	917 ± 137
**Meteorological stations**			
Station name	Pully	Buchs - Aarau	Schaffhausen
Latitude (N)	46° 30′ 44.218″	47° 23′ 03.763″	47° 41′ 23.401″
Longitude (E)	6° 40′ 03.058″	8° 04′ 46.341″	8° 37′ 12.571″
Elevation (m a.s.l.)	456	386	438
Distance to site (km)	5	13.5	3.2

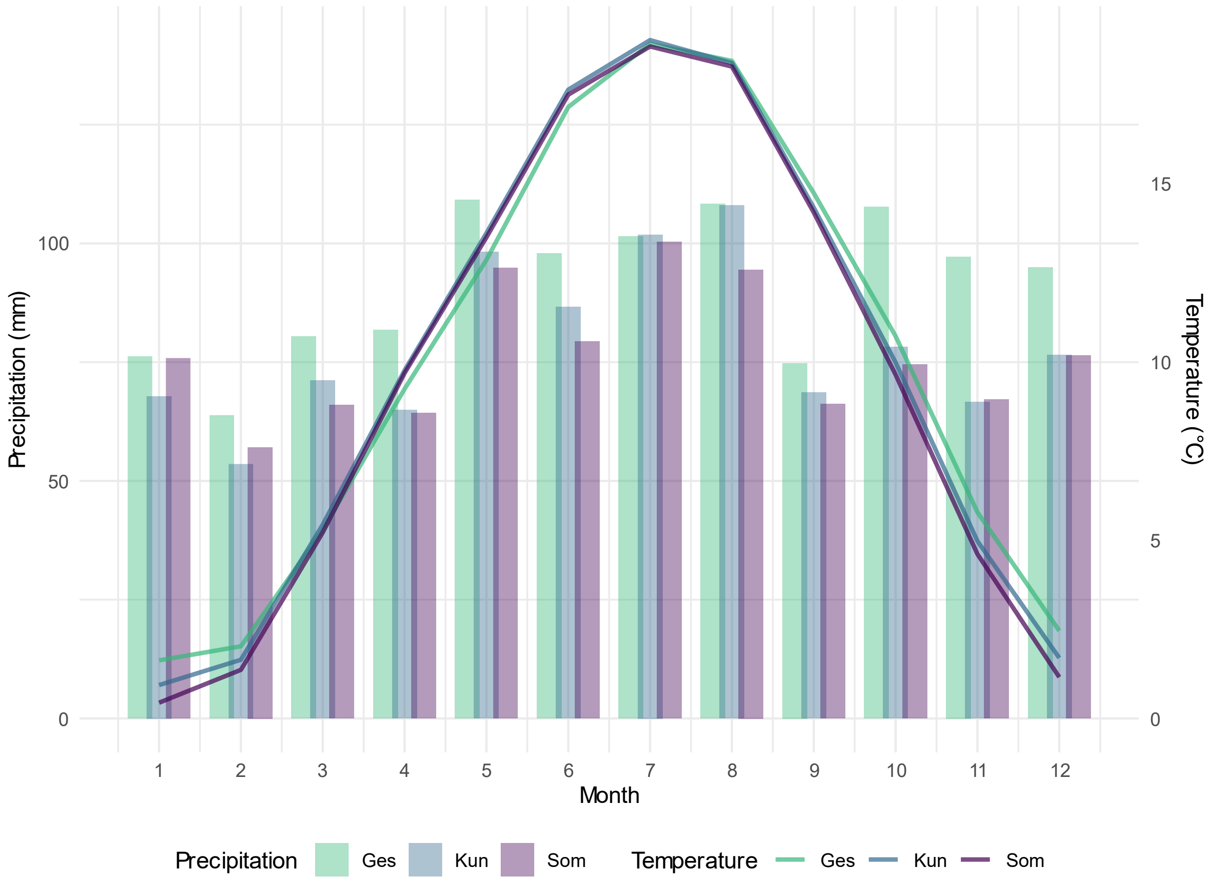

Supplementary Figure 1. Monthly average temperature and precipitation sum over the period 2000–2020 at the study sites.

Supplementary Table 2. Mean inter-series correlation ($\bar{r}$) and EPS (expressed population signal) calculated for the period 2000–2020 for each tree-ring variable, species and site. Eight trees are included per species and site.

		**δ^{18}O**		**δ^{2}H**		**TRW**	
**Site**	**Species**	$\bar{r}$	EPS	$\bar{r}$	EPS	$\bar{r}$	EPS
Ges	Silver fir	0.515	0.895	0.299	0.774	0.230	0.705
	Douglas-fir	0.513	0.894	0.388	0.836	0.302	0.776
Kun	Silver fir	0.453	0.869	0.112	0.502	0.378	0.829
	Douglas-fir	0.638	0.934	0.314	0.785	0.298	0.773
Som	Silver fir	0.473	0.878	0.211	0.682	0.043	0.265
	Douglas-fir	0.725	0.955	0.417	0.851	0.308	0.781

Supplementary Equation 1

We calculated the daily vapor pressure deficit (VPD; kPa) after Eq. 11 and 17 in Allen et al. (1998):

$$VPD = SVP - AVP$$

with SVP (saturated vapor pressure; kPa):

$$SVP = (SVP_{Tmin} + SVP_{Tmax})/2$$

with SVP_{Tmin} the saturated vapor pressure at daily minimum temperature:

$$SVP_{Tmin} = 0.6108 \cdot \exp\left(\frac{17.27 \cdot T_{min}}{T_{min} + 237.3}\right)$$

and SVP_{Tmax} the saturated vapor pressure at daily maximum temperature:

$$SVP_{Tmax} = 0.6108 \cdot \exp\left(\frac{17.27 \cdot T_{max}}{T_{max} + 237.3}\right)$$

and with AVP (actual vapor pressure; kPa):

$$AVP = \left[SVP_{Tmin} \cdot \frac{RH_{max}}{100} + SVP_{Tmax} \cdot \frac{RH_{min}}{100}\right]/2$$

with T_{min} (T_{max}) the daily minimum (maximum) temperature (°C) and RH_{min} (RH_{max}) the daily minimum (maximum) relative humidity (%).

Supplementary Table 3. Summary table of the mixed-effects model explaining the δ^{18}O-δ^{2}H relationship at the individual level (see Figure 3a-b). Individual data were used in the models, and the variables x correspond to the tree identity and their interaction with δ^{18}O. Significant P-values (≤ 0.05) are highlighted in bold.

Variable y	Variable x	Estimate	Standard error	P-value
**Silver fir**				
δ^{2}H	Intercept	-56.75	90.16	0.53
	δ^{18}O	0.97	3.05	0.75
	GesA04	11.00	133.24	0.93
	GesA06	-22.67	127.65	0.86
	GesA08	196.63	119.97	0.10
	GesA09	-1.80	120.17	0.99
	GesA14	77.34	125.09	0.54
	GesA17	123.12	125.35	0.33
	GesA18	111.79	126.35	0.38
	KunA05	137.05	103.36	0.19
	KunA06	-12.12	111.13	0.91
	KunA07	224.99	130.68	0.09
	KunA08	370.08	116.24	**<0.01**
	KunA10	14.61	113.52	0.90
	KunA11	309.38	119.80	**0.01**
	KunA14	257.28	114.47	**0.03**
	KunA15	168.64	119.17	0.16
	SomA02	47.96	111.15	0.67
	SomA03	178.97	107.79	0.10
	SomA06	-42.10	120.22	0.73
	SomA07	72.10	101.26	0.48
	SomA08	116.41	99.43	0.24
	SomA14	95.81	101.52	0.35
	SomA17	-45.01	103.92	0.67
	SomA19	-34.55	115.41	0.76
	δ^{18}O:GesA04	-0.64	4.58	0.89
	δ^{18}O:GesA06	0.21	4.33	0.96
	δ^{18}O:GesA08	-8.11	4.13	**0.05**
	δ^{18}O:GesA09	-0.04	4.15	0.99
	δ^{18}O:GesA14	-3.10	4.21	0.46
	δ^{18}O:GesA17	-4.48	4.29	0.30
	δ^{18}O:GesA18	-3.74	4.33	0.39
	δ^{18}O:KunA05	-4.88	3.56	0.17
	δ^{18}O:KunA06	1.55	3.85	0.69
	δ^{18}O:KunA07	-7.75	4.49	0.09
	δ^{18}O:KunA08	-13.50	3.98	**<0.01**
	δ^{18}O:KunA10	-1.04	3.90	0.79
	δ^{18}O:KunA11	-10.41	4.14	**0.01**
	δ^{18}O:KunA14	-8.54	3.93	**0.03**

	δ^{18}O:KunA15	-5.77	4.02	0.15
	δ^{18}O:SomA02	-1.90	3.78	0.61
	δ^{18}O:SomA03	-6.76	3.67	0.07
	δ^{18}O:SomA06	0.59	4.10	0.89
	δ^{18}O:SomA07	-2.72	3.45	0.43
	δ^{18}O:SomA08	-4.58	3.44	0.18
	δ^{18}O:SomA14	-3.91	3.52	0.27
	δ^{18}O:SomA17	0.83	3.54	0.81
	δ^{18}O:SomA19	0.48	3.87	0.90
**Douglas-fir**				
δ^{2}H	Intercept	117.08	61.11	0.06
	δ^{18}O	-5.83	2.17	**0.01**
	GesP05	-151.01	98.93	0.13
	GesP06	-6.31	96.11	0.95
	GesP09	-119.16	78.65	0.13
	GesP12	-184.50	84.71	**0.03**
	GesP17	-314.94	92.04	**<0.01**
	GesP18	-70.24	102.48	0.49
	GesP19	-115.24	117.11	0.33
	KunP02	-259.08	98.80	**0.01**
	KunP03	-165.74	100.71	0.10
	KunP05	-386.51	102.94	**<0.01**
	KunP13	-157.38	86.99	0.07
	KunP14	-239.96	92.34	**0.01**
	KunP16	-193.54	88.22	**0.03**
	KunP18	-231.21	99.00	**0.02**
	KunP19	-245.59	92.87	**0.01**
	SomP02	-247.64	86.38	**<0.01**
	SomP05	-177.13	97.23	0.07
	SomP06	-344.20	98.71	**<0.01**
	SomP08	-302.30	89.96	**<0.01**
	SomP10	-412.69	122.71	**<0.01**
	SomP13	-254.03	88.21	**<0.01**
	SomP14	-242.90	100.94	**0.02**
	SomP20	-310.03	105.24	**<0.01**
	δ^{18}O:GesP05	5.62	3.52	0.11
	δ^{18}O:GesP06	0.95	3.29	0.77
	δ^{18}O:GesP09	4.89	2.77	0.08
	δ^{18}O:GesP12	6.35	2.98	**0.03**
	δ^{18}O:GesP17	11.19	3.19	**<0.01**
	δ^{18}O:GesP18	3.03	3.56	0.39
	δ^{18}O:GesP19	4.67	4.13	0.26
	δ^{18}O:KunP02	9.00	3.43	**0.01**

$\delta^{18}O$:KunP03	6.72	3.57	0.06
$\delta^{18}O$:KunP05	13.64	3.57	**<0.01**
$\delta^{18}O$:KunP13	6.05	3.05	**0.05**
$\delta^{18}O$:KunP14	8.66	3.20	**0.01**
$\delta^{18}O$:KunP16	6.52	3.09	**0.04**
$\delta^{18}O$:KunP18	8.88	3.47	**0.01**
$\delta^{18}O$:KunP19	8.29	3.28	**0.01**
$\delta^{18}O$:SomP02	7.58	3.03	**0.01**
$\delta^{18}O$:SomP05	6.41	3.43	0.06
$\delta^{18}O$:SomP06	11.55	3.47	**<0.01**
$\delta^{18}O$:SomP08	10.05	3.12	**<0.01**
$\delta^{18}O$:SomP10	14.39	4.27	**<0.01**
$\delta^{18}O$:SomP13	9.46	3.09	**<0.01**
$\delta^{18}O$:SomP14	8.83	3.53	**0.01**
$\delta^{18}O$:SomP20	10.67	3.64	**<0.01**

Supplementary Table 4. Summary table of the mixed-effects model explaining the between $\delta^{18}O$-$\delta^{2}H$ relationship at the site level (see Figure 3c-d). Site chronologies (i.e., average of the eight trees per year) were used in the models. Significant P-values (≤ 0.05) are highlighted in bold.

Variable y	Variable x	Estimate	Standard error	P-value
**Silver fir**				
$\delta^{2}H$	Intercept	-50.585	32.879	0.129
	$\delta^{18}O$	0.542	1.137	0.635
**Douglas-fir**				
$\delta^{2}H$	Intercept	-166.004	31.887	**<0.001**
	$\delta^{18}O$	4.221	1.094	**<0.001**

Supplementary Table 5. Summary table of the additive mixed-models analyzing $\delta^{18}O$ and $\delta^{2}H$ in relation to tree-ring width (TRW) for silver fir and Douglas-fir (see Figure 4). Estimate/Effective degrees of freedom (EDF) are given for the linear intercept and the smoothing terms, respectively, and similarly for standard error and F-values. Significant P-values (≤ 0.05) are highlighted in bold.

**Silver fir**				
**Variable y**	**Variable x**	**Estimate/EDF**	**SE/F-value**	**P-value**
$\delta^{18}O$	Intercept	28.715	0.32	**<0.001**
	TRW:site Ges	1.000	21.34	**<0.001**
	TRW:site Kun	2.672	31.42	**<0.001**
	TRW:site Som	2.799	47.73	**<0.001**
	Tree identity	19.675	17.70	**<0.001**
504 observations; Adjusted R^2: 0.58; Deviance explained: 60.3%				
$\delta^{2}H$	Intercept	-32.406	3.610	**<0.001**
	TRW:site Ges	2.242	26.66	**<0.001**
	TRW:site Kun	2.106	58.26	**<0.001**
	TRW:site Som	2.835	34.42	**<0.001**
	Tree identity	19.796	18.35	**<0.001**
504 observations; Adjusted R^2: 0.70; Deviance explained: 71.7%				

**Douglas-fir**				
**Variable y**	**Variable x**	**Estimate/EDF**	**SE/F-value**	**P-value**
$\delta^{18}O$	Intercept	28.936	0.15	**<0.001**
	TRW:site Ges	1.000	12.49	**<0.001**
	TRW:site Kun	1.053	3.37	0.058
	TRW:site Som	1.000	3.50	0.062
	Tree identity	16.698	4.41	**<0.001**
504 observations; Adjusted R^2: 0.21; Deviance explained: 24.8%				
$\delta^{2}H$	Intercept	-41.621	4.02	**<0.001**
	TRW:site Ges	2.640	30.28	**<0.001**
	TRW:site Kun	1.000	5.51	**0.019**
	TRW:site Som	1.600	10.91	**<0.001**
	Tree identity	20.320	36.10	**<0.001**
504 observations; Adjusted R^2: 0.71; Deviance explained: 72.7%				

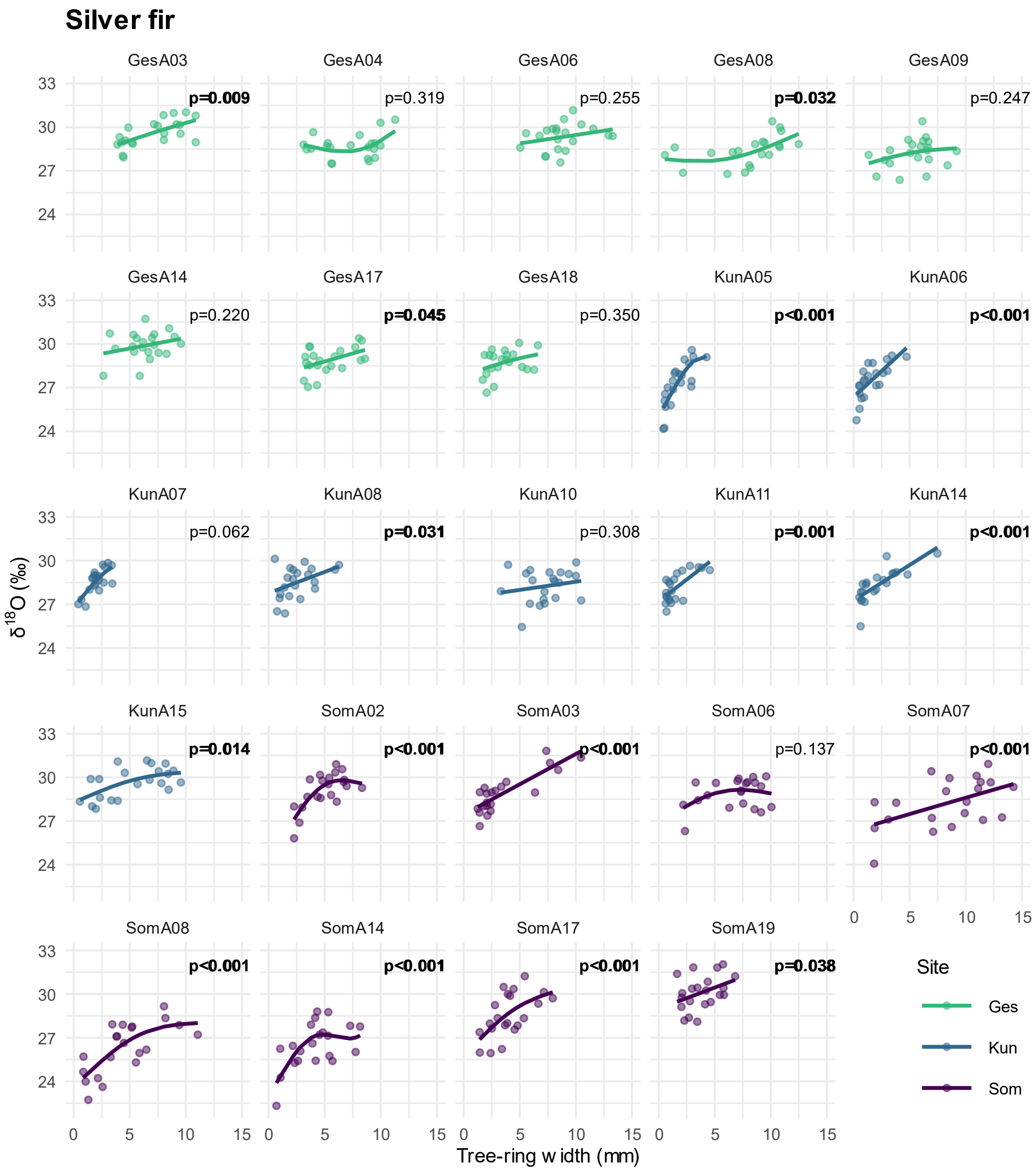

Supplementary Figure 2. Relationship between δ^{18}O and TRW for each individual silver fir tree at the three study sites. The points show the raw data of the individual trees. The curves are fitted with additive mixed models following model 4 (Supporting Information). For brevity, we do not include the complete output of the model, but the P-values for each tree are indicated in the top right corner or each subplot with significant P-values (≤ 0.05) highlighted in bold. The model was based on 504 observations, with adjusted $R^2 = 0.59$ and deviance explained = 63.1%.

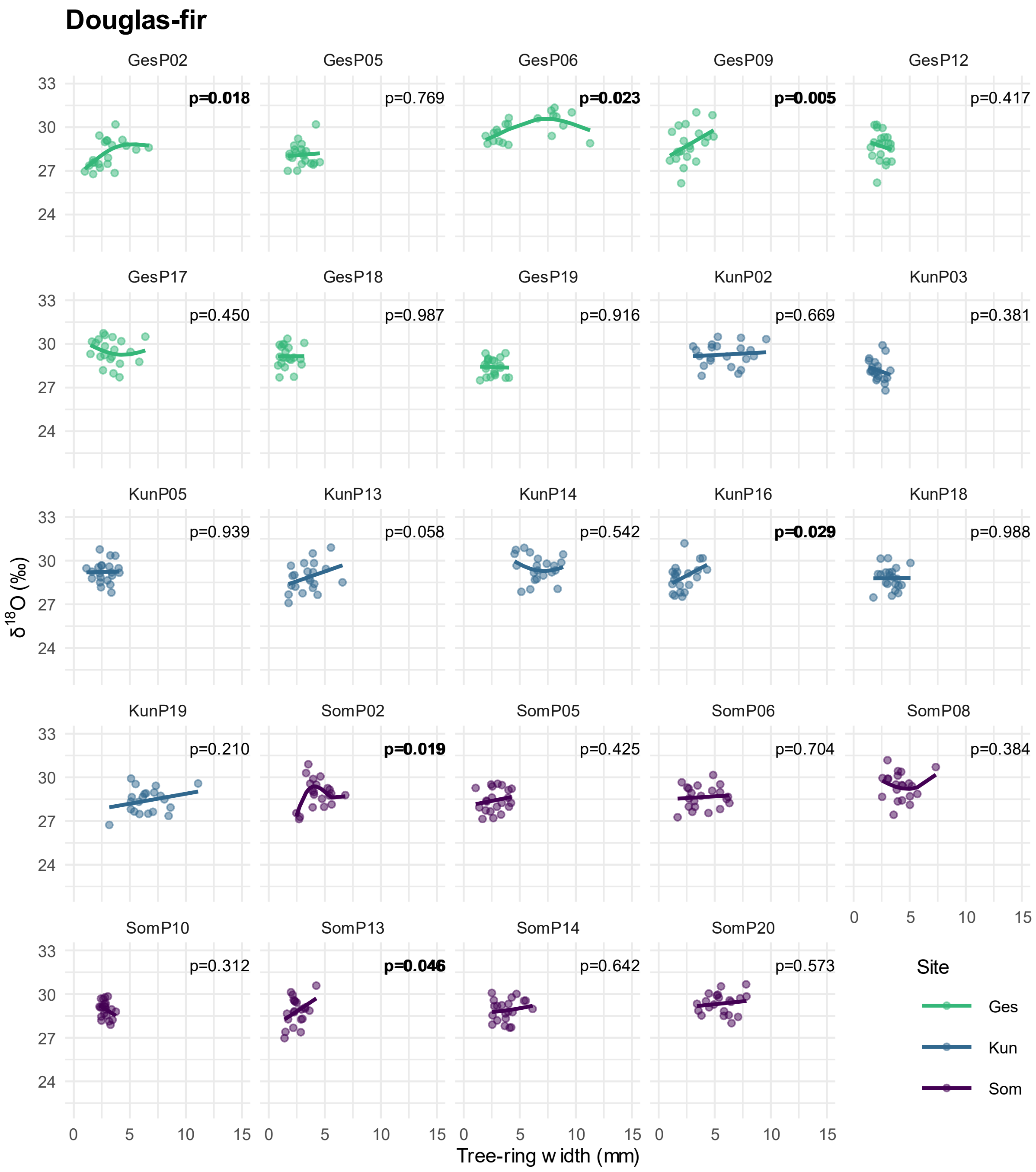

Supplementary Figure 3. Relationship between δ^{18}O and TRW for each individual Douglas-fir tree at the three study sites. The points show the raw data of the individual trees. The curves are fitted with additive mixed models following model 4 (Supporting Information). For brevity, we do not include the complete output of the model, but the P-values for each tree are indicated in the top right corner or each subplot with significant P-values (≤ 0.05) highlighted in bold. The model was based on 504 observations, with adjusted R^2 = 0.25 and deviance explained = 32.9%.

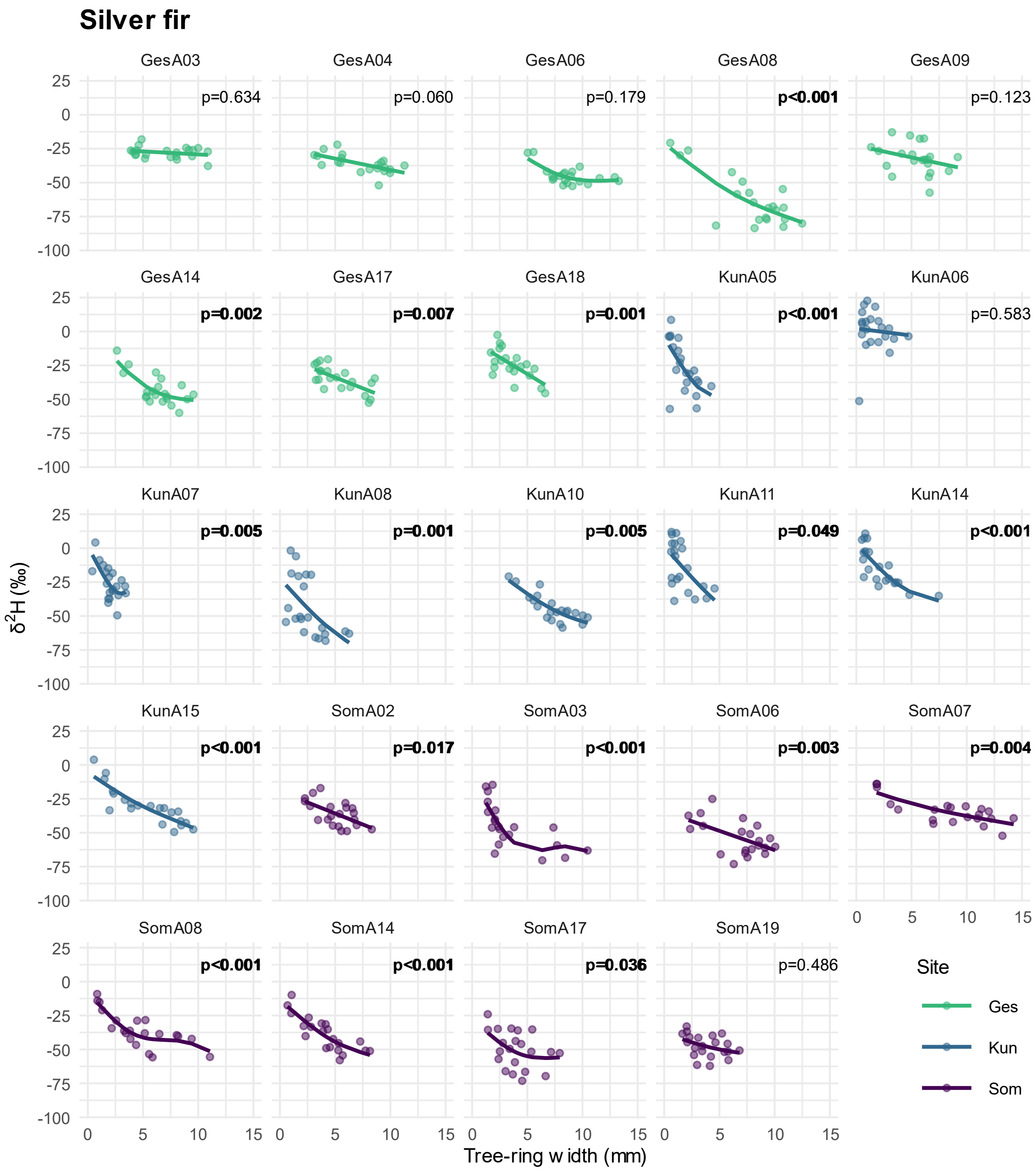

Supplementary Figure 4. Relationship between δ^2H and TRW for each individual silver fir tree at the three study sites. The points show the raw data of the individual trees. The curves are fitted with additive mixed models following model 4 (Supporting Information). For brevity, we do not include the complete output of the model, but the P-values for each tree are indicated in the top right corner or each subplot with significant P-values (≤ 0.05) highlighted in bold. The model was based on 504 observations, with adjusted $R^2 = 0.71$ and deviance explained = 74.3%.

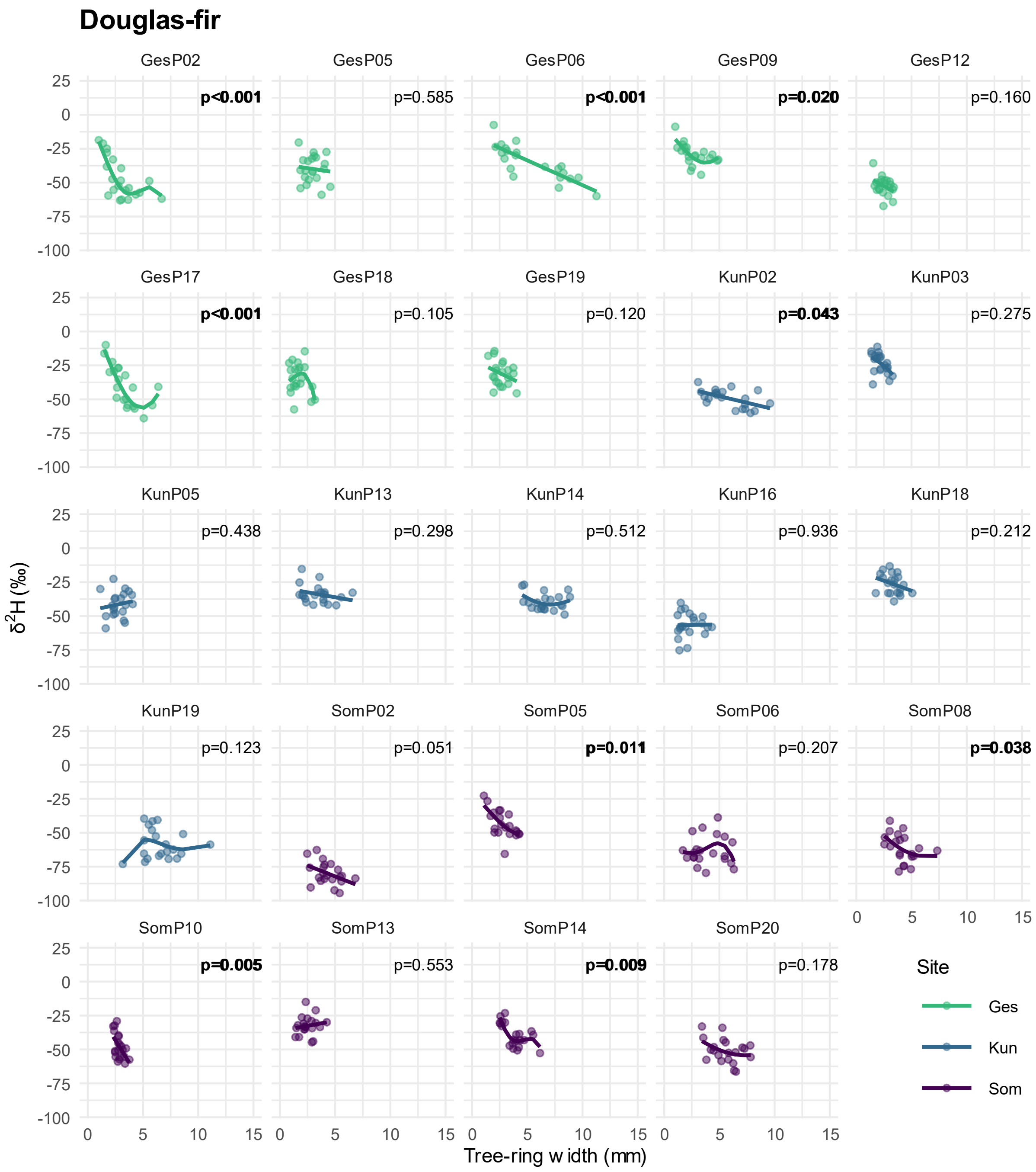

Supplementary Figure 5. Relationship between δ^2H and TRW for each individual Douglas-fir tree at the three study sites. The points show the raw data of the individual trees. The curves are fitted with additive mixed models following model 4 (Supporting Information). For brevity, we do not include the complete output of the model, but the P-values for each tree are indicated in the top right corner or each subplot with significant P-values (≤ 0.05) highlighted in bold. The model was based on 504 observations, with adjusted R^2 = 0.75 and deviance explained = 77.7%.

Supplementary Table 6. Summary of the mixed-effects models analyzing $\delta^{18}O$ and $\delta^{2}H$ in relation to tree estimated age (see Figure 5). Significant P-values (≤ 0.05) are highlighted in bold. One model was calculated per isotope variable, species and site.

**Silver fir**				
**Variable y**	**Variable x**	**Estimate**	**Standard error**	**P-value**
**$\delta^{18}O$**				
Ges	Intercept	29.225	0.468	**<0.001**
	Estimated age	-0.007	0.011	0.528
Kun	Intercept	34.161	1.214	**<0.001**
	Estimated age	-0.060	0.011	**<0.001**
Som	Intercept	28.286	1.003	**<0.001**
	Estimated age	0.001	0.016	0.964
**$\delta^{2}H$**				
Ges	Intercept	-62.379	5.910	**<0.001**
	Estimated age	0.634	0.125	**<0.001**
Kun	Intercept	-93.569	17.298	**<0.001**
	Estimated age	0.707	0.170	**<0.001**
Som	Intercept	-34.867	7.538	**<0.001**
	Estimated age	-0.137	0.123	0.267

**Douglas-fir**				
**Variable y**	**Variable x**	**Estimate**	**Standard error**	**P-value**
**$\delta^{18}O$**				
Ges	Intercept	29.897	1.177	**<0.001**
	Estimated age	-0.010	0.011	0.360
Kun	Intercept	27.150	0.995	**<0.001**
	Estimated age	0.017	0.010	0.079
Som	Intercept	28.830	0.566	**<0.001**
	Estimated age	0.001	0.006	0.868
**$\delta^{2}H$**				
Ges	Intercept	-28.752	14.762	**0.053**
	Estimated age	-0.097	0.140	0.490
Kun	Intercept	-42.896	11.118	**<0.001**
	Estimated age	0.017	0.102	0.868
Som	Intercept	-74.632	10.798	**<0.001**
	Estimated age	0.253	0.109	**0.021**

Supplementary Table 7. Summary of the mixed-effects models analyzing $\delta^{18}O$ and $\delta^{2}H$ in relation to crown volume for silver fir and Douglas-fir (see Figure 6). One model was calculated per isotope and per species. Significant P-values (≤ 0.05) are highlighted in bold.

**Silver fir**				
**Variable y**	**Variable x**	**Estimate**	**Standard error**	**P-value**
$\delta^{18}O$	Intercept	29.985	1.318	**<0.001**
	Crown volume	-0.006	0.004	0.132
$\delta^{2}H$	Intercept	-39.452	11.980	**0.004**
	Crown volume	0.041	0.025	0.121

**Douglas-fir**				
**Variable y**	**Variable x**	**Estimate**	**Standard error**	**P-value**
$\delta^{18}O$	Intercept	28.775	0.392	**<0.001**
	Crown volume	0.001	0.001	0.330
$\delta^{2}H$	Intercept	-48.052	9.218	**<0.001**
	Crown volume	0.012	0.011	0.299

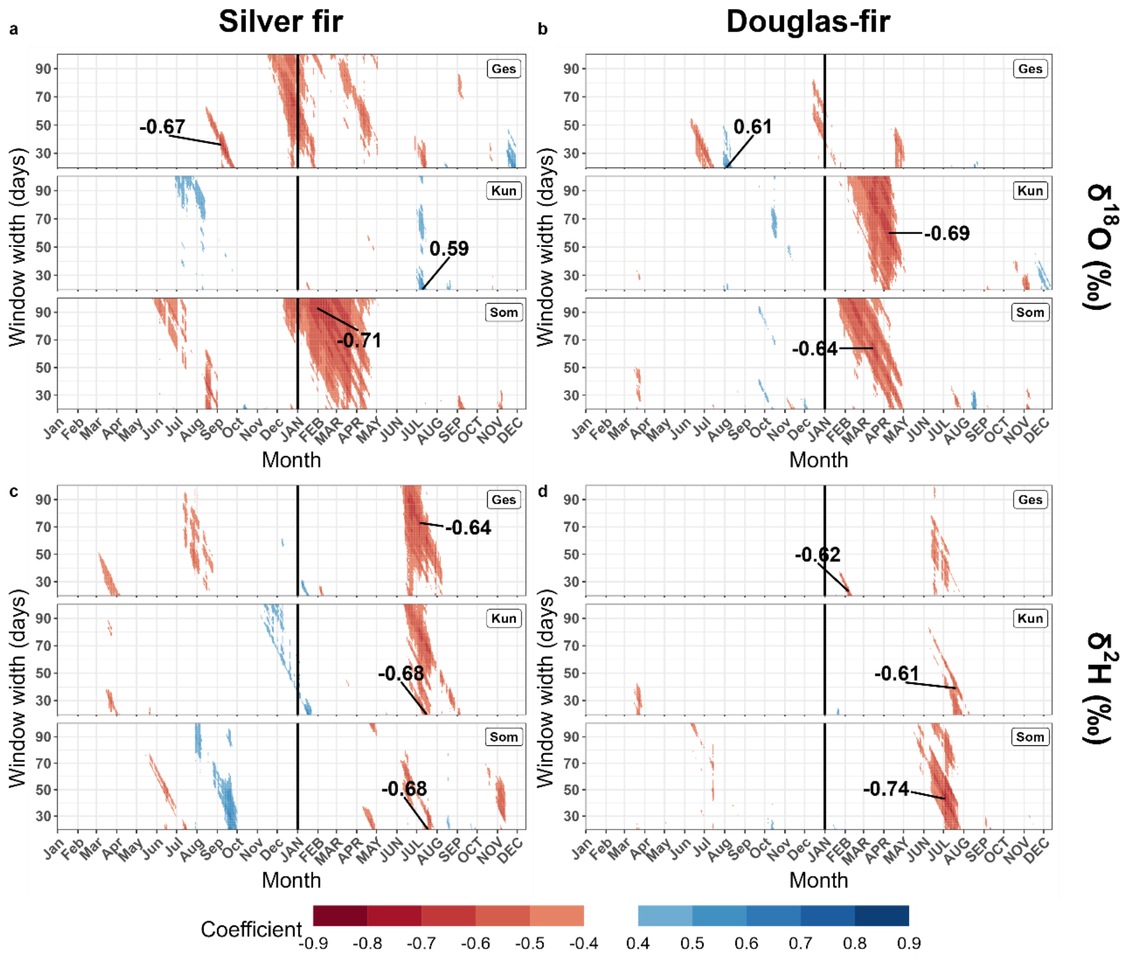

Supplementary Figure 6. Moving-window correlations between daily precipitation sum and tree-ring chronologies of (a) $\delta^{18}O$ of silver fir, (b) $\delta^{18}O$ of Douglas-fir, (c) $\delta^{2}H$ of silver fir and (d) $\delta^{2}H$ of Douglas-fir for the period 2000–2020 at the three study sites (labels on the top right corners). Only significant correlations are shown (P-value ≤ 0.05). The day of the month on the x-axis corresponds to the beginning of the window width (e.g., a point at 60 days on the y-axis and the beginning of July on the x-axis corresponds to a correlation with the climate data of 60 days starting at the beginning of July, i.e., covering July and August). Months in lowercase denote the months of the year prior to tree-ring formation. Numbers in bold indicate the highest correlation per site.

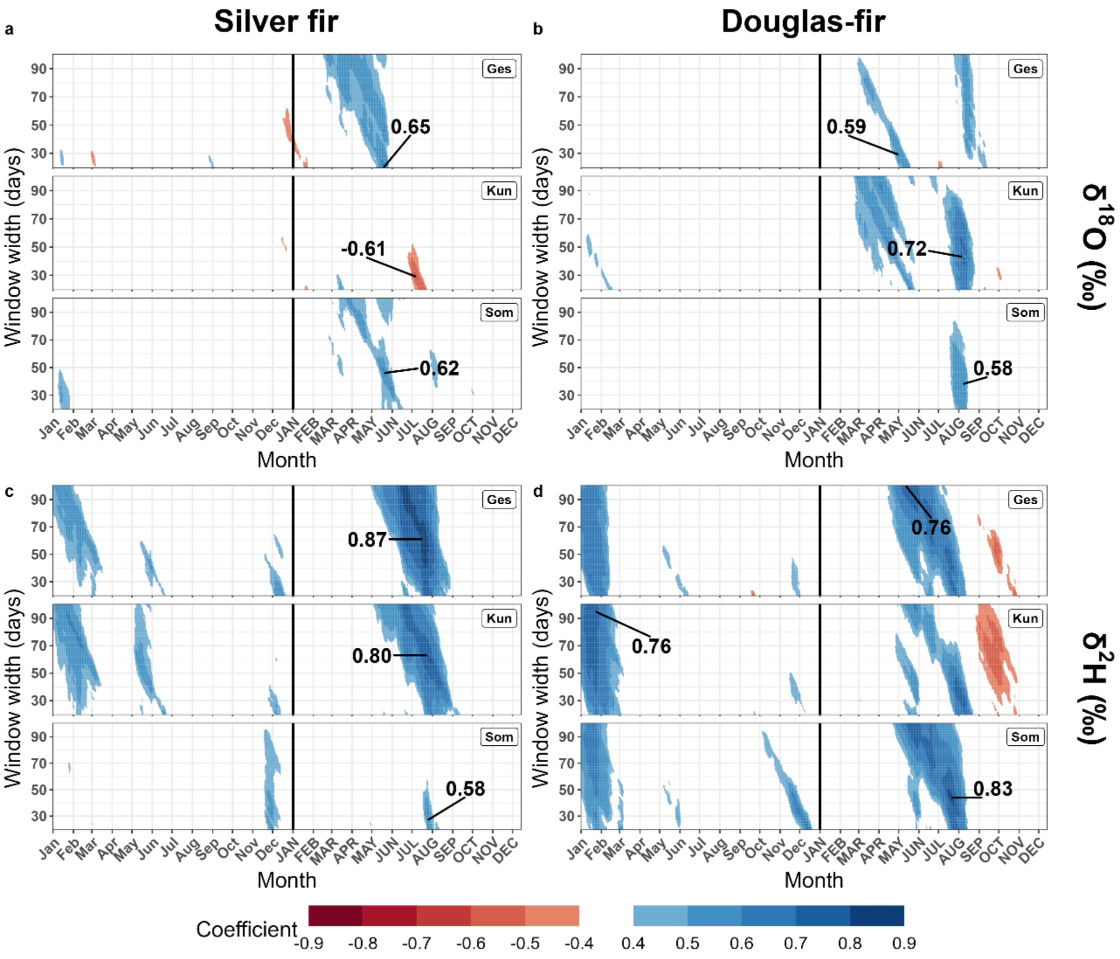

Supplementary Figure 7. Moving-window correlations between daily average temperature and tree-ring chronologies of (a) δ^{18}O of silver fir, (b) δ^{18}O of Douglas-fir, (c) δ^{2}H of silver fir and (d) δ^{2}H of Douglas-fir for the period 2000–2020 at the three study sites (labels on the top right corners). Only significant correlations are shown (P-value $\leq$ 0.05). The day of the month on the x-axis corresponds to the beginning of the window width (e.g., a point at 60 days on the y-axis and the beginning of July on the x-axis corresponds to a correlation with the climate data of 60 days starting at the beginning of July, i.e., covering July and August). Months in lowercase denote the months of the year prior to tree-ring formation. Numbers in bold indicate the highest correlation per site.

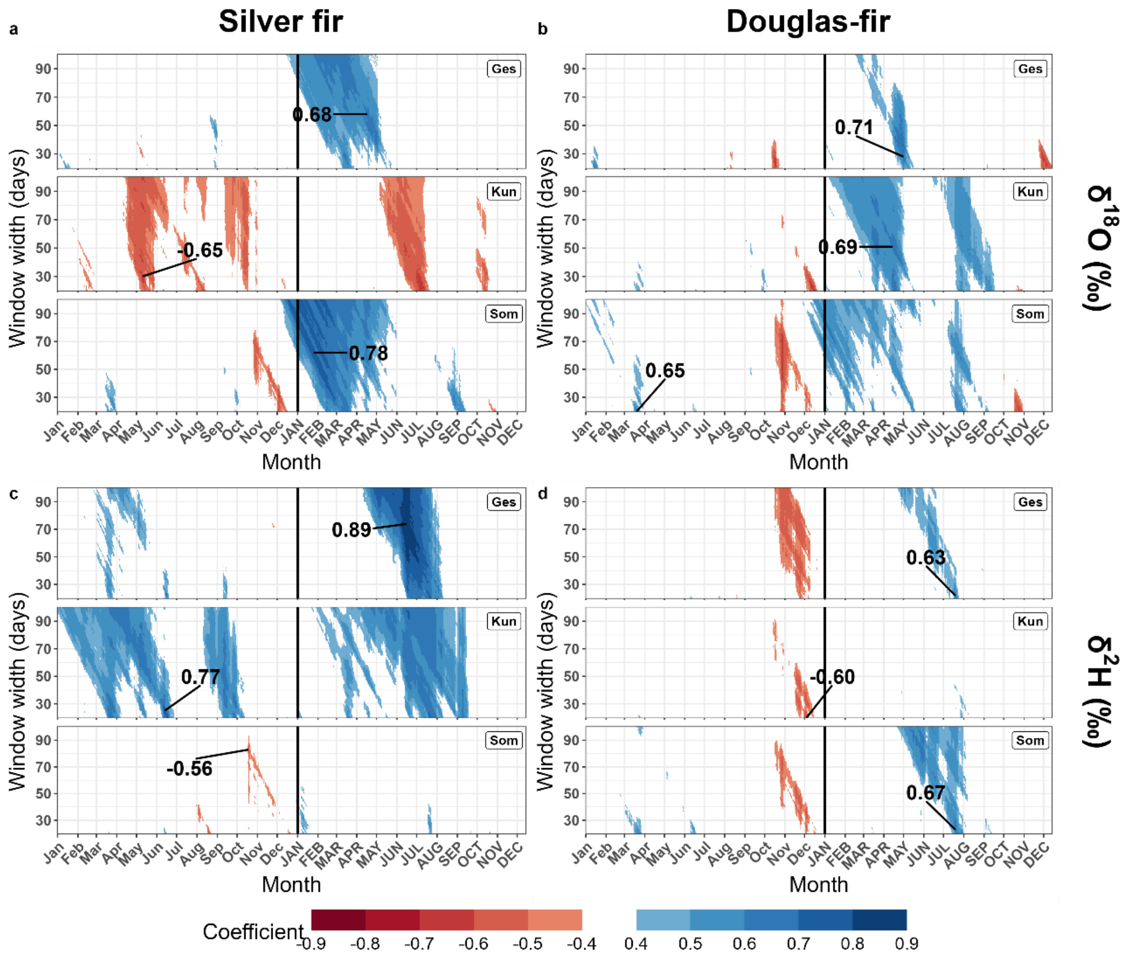

Supplementary Figure 8. Moving-window correlations between daily average global radiation and tree-ring chronologies of (a) δ^{18}O of silver fir, (b) δ^{18}O of Douglas-fir, (c) δ^{2}H of silver fir and (d) δ^{2}H of Douglas-fir for the period 2000–2020 at the three study sites (labels on the top right corners). Only significant correlations are shown (P-value $\leq$ 0.05). The day of the month on the x-axis corresponds to the beginning of the window width (e.g., a point at 60 days on the y-axis and the beginning of July on the x-axis corresponds to a correlation with the climate data of 60 days starting at the beginning of July, i.e., covering July and August). Months in lowercase denote the months of the year prior to tree-ring formation. Numbers in bold indicate the highest correlation per site.

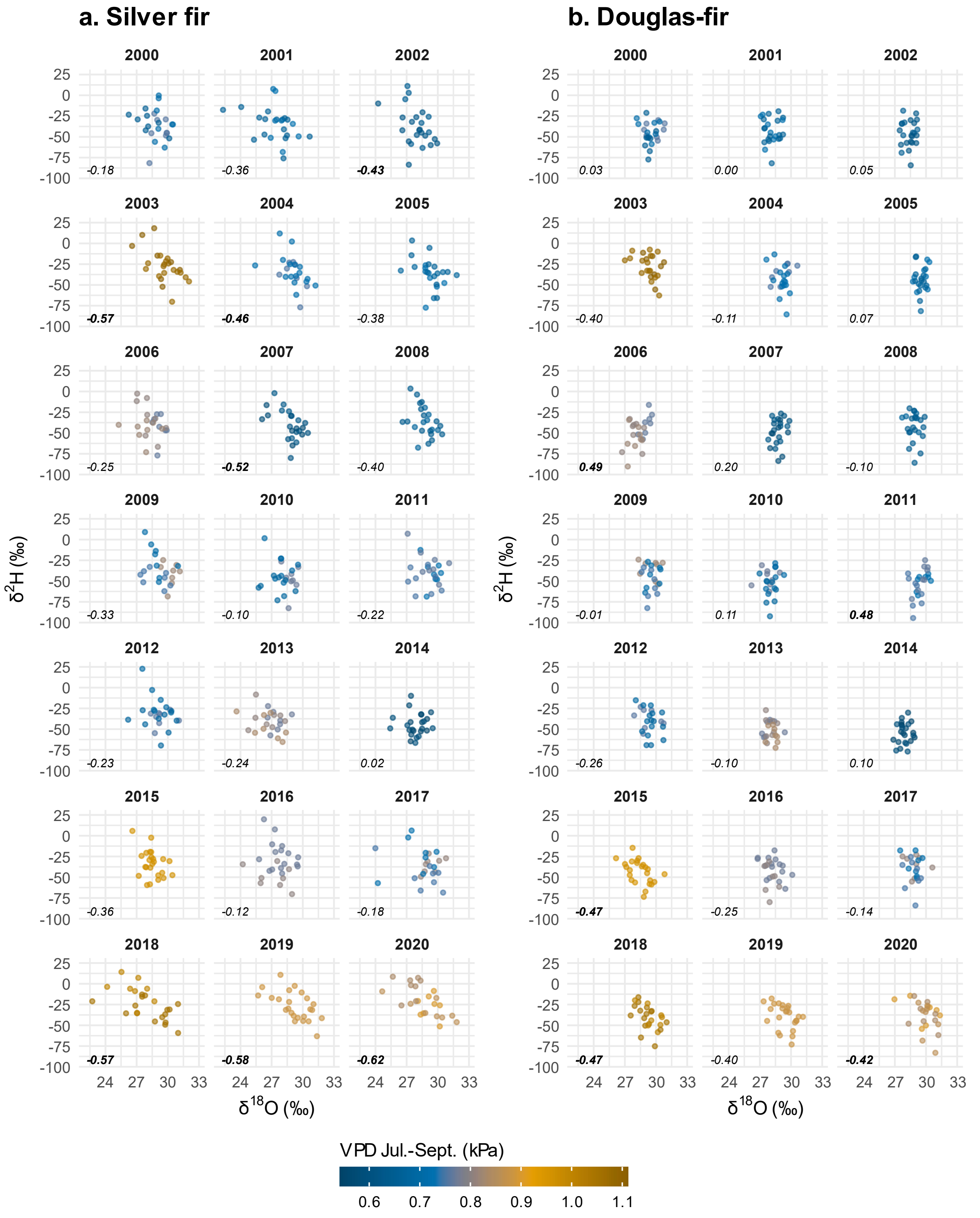

Supplementary Figure 9. δ^2H against δ^{18}O per year for (a) silver fir and (b) Douglas-fir. Each panel includes data from eight individual trees at the three study sites. VPD stands for vapor pressure deficit, calculated for the period July to September. The correlation coefficient is indicated at the bottom left corner of each panel. Significant P-values (≤ 0.05) are highlighted in bold.

Supplementary Table 8. Summary table of the linear model explaining the relationship between summer VPD (average July-September) and the $\delta^{18}O$-$\delta^{2}H$ correlation coefficients presented in Figure 8. Significant P-values (≤ 0.05) are highlighted in bold.

Variable y	Variable x	Estimate	Standard error	P-value
**Silver fir**				
Corr. $\delta^{18}O$-$\delta^{2}H$	Intercept	0.154	0.207	0.464
	VPD summer	-0.640	0.265	**0.026**
**Douglas-fir**				
Corr. $\delta^{18}O$-$\delta^{2}H$	Intercept	0.931	0.285	**0.004**
	VPD summer	-1.310	0.365	**0.002**

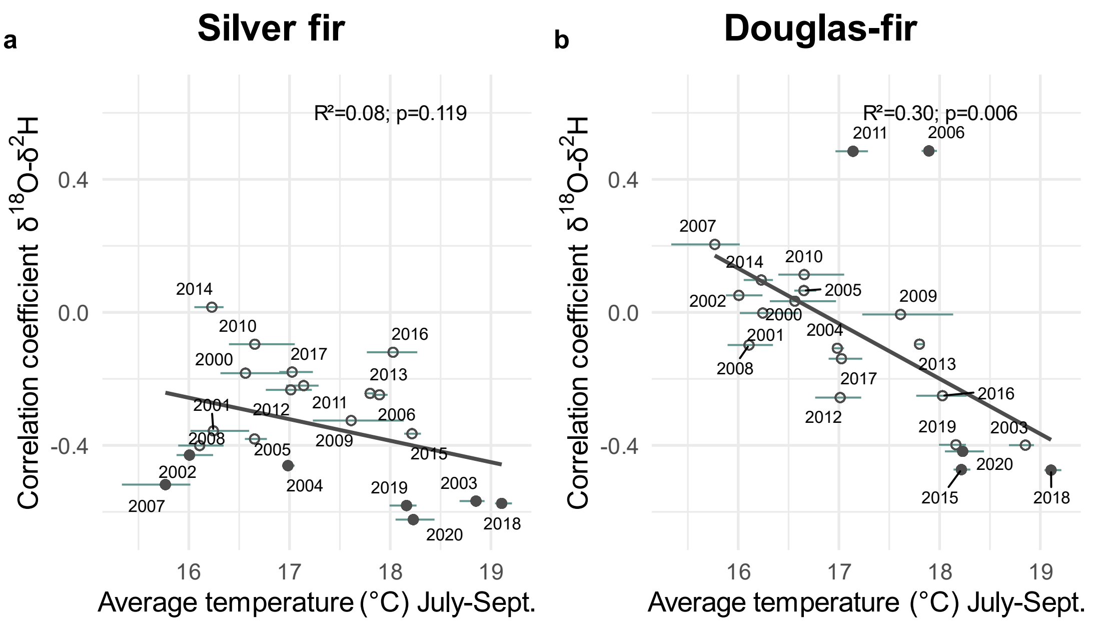

Supplementary Figure 10. Relationship between the δ^{18}O-δ^{2}H correlation coefficients and the average summer temperature (July to September) for (a) silver fir and (b) Douglas-fir. The filled circles and the empty circles represent significant and non-significant values of Pearson's correlation coefficients ($P \leq 0.05$), respectively. Each correlation coefficient includes 24 pairs of δ^{18}O-δ^{2}H values (three sites pooled with eight trees each). The dark solid line on each panel shows the fitted relationships following model 8 (Supporting Information). The horizontal lines overlapping each circle show the range of temperature values for the three sites for a given year. Adjusted R^2 and P-values are shown for each fitted model.

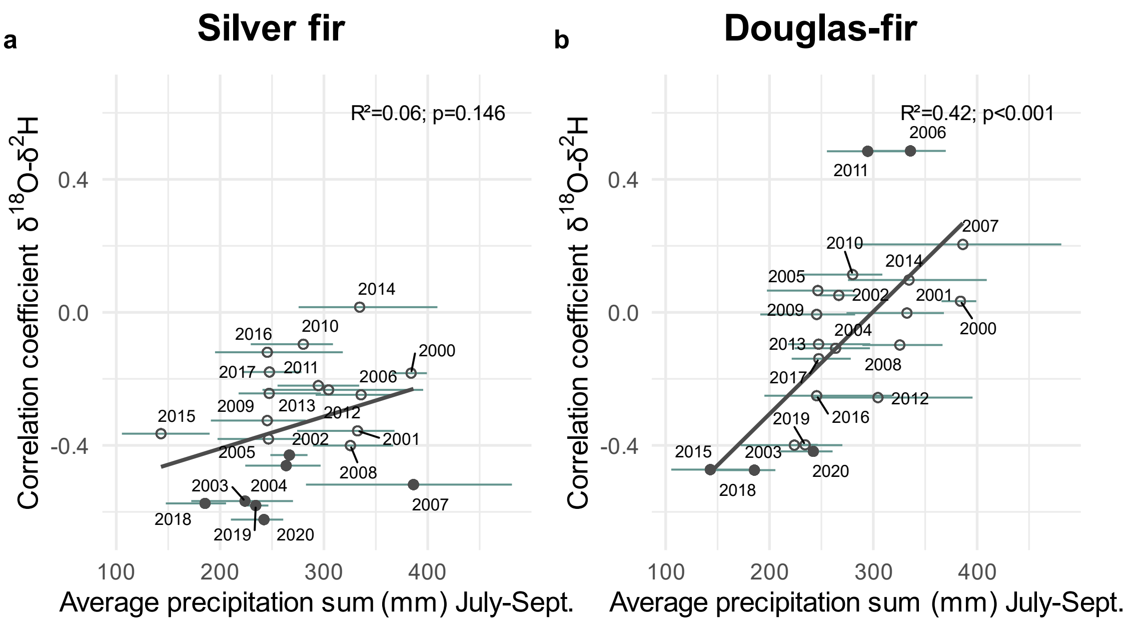

Supplementary Figure 11. Relationship between the δ^{18}O-δ^{2}H correlation coefficients and the average summer precipitation sum (July to September) for (a) silver fir and (b) Douglas-fir. The filled circles and the empty circles represent significant and non-significant values of Pearson's correlation coefficients ($P \leq 0.05$), respectively. Each correlation coefficient includes 24 pairs of δ^{18}O-δ^{2}H values (three sites pooled with eight trees each). The dark solid line on each panel shows the fitted relationships following model 8 (Supporting Information). The horizontal lines overlapping each circle show the range of precipitation sum values for the three sites for a given year. Adjusted R^2 and P-values are shown for each fitted model.

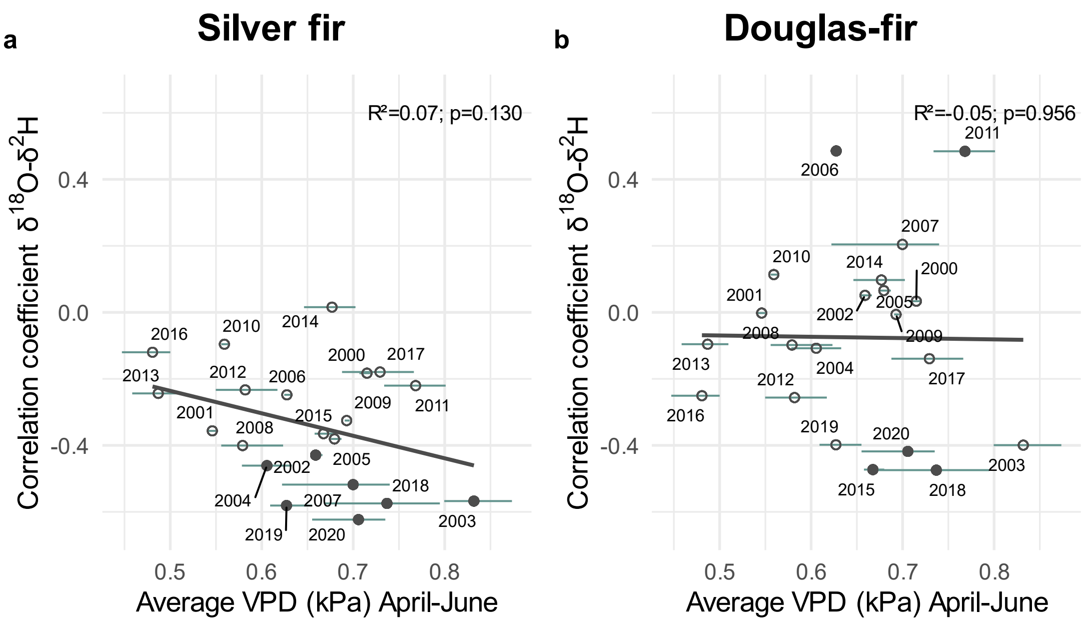

Supplementary Figure 12. Relationship between the δ^{18}O-δ^{2}H correlation coefficients and the average spring vapor pressure deficit (VPD; April to June) for (a) silver fir and (b) Douglas-fir. The filled circles and the empty circles represent significant and non-significant values of Pearson's correlation coefficients ($P \leq 0.05$), respectively. Each correlation coefficient includes 24 pairs of δ^{18}O-δ^{2}H values (three sites pooled with eight trees each). The dark solid line on each panel shows the fitted relationships following model 8 (Supporting Information). The horizontal lines overlapping each circle show the range of VPD values for the three sites for a given year. Adjusted R^2 and P-values are shown for each fitted model.

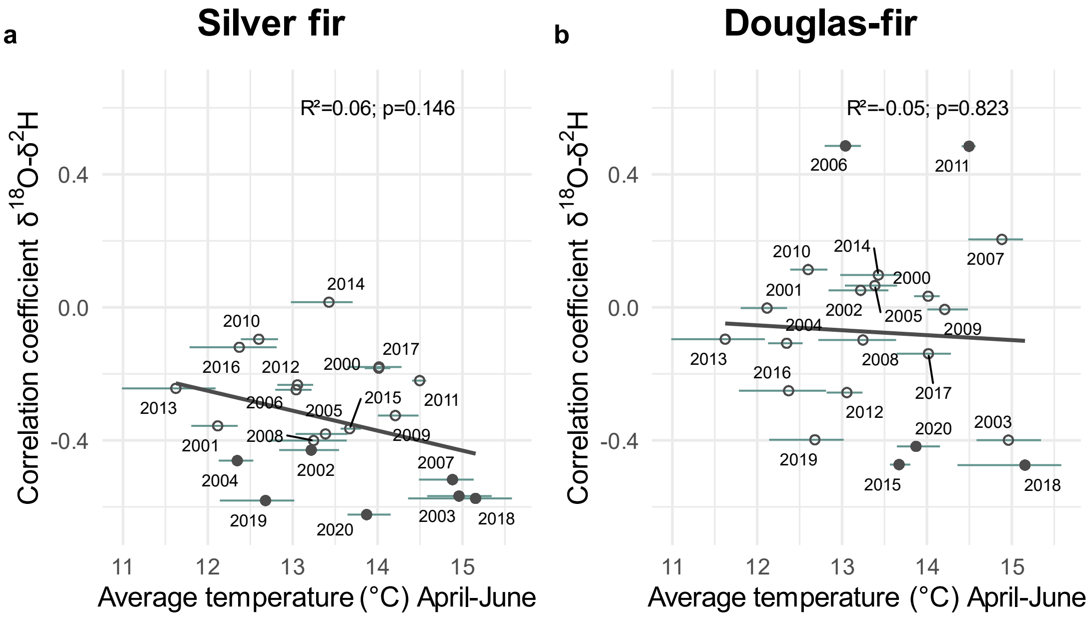

Supplementary Figure 13. Relationship between the δ^{18}O-δ^{2}H correlation coefficients and the average spring temperature (April to June) for (a) silver fir and (b) Douglas-fir. The filled circles and the empty circles represent significant and non-significant values of Pearson's correlation coefficients ($P \leq 0.05$), respectively. Each correlation coefficient includes 24 pairs of δ^{18}O-δ^{2}H values (three sites pooled with eight trees each). The dark solid line on each panel shows the fitted relationships following model 8 (Supporting Information). The horizontal lines overlapping each circle show the range of temperature values for the three sites for a given year. Adjusted R^2 and P-values are shown for each fitted model.

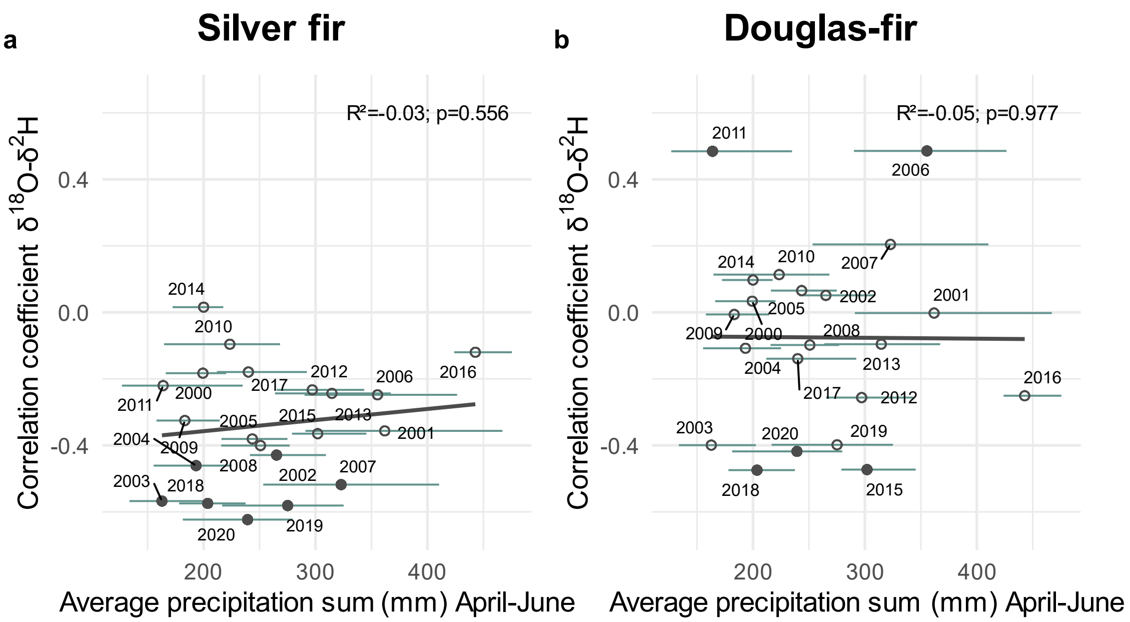

Supplementary Figure 14. Relationship between the δ^{18}O-δ^{2}H correlation coefficients and the average spring precipitation sum (April to June) for (a) silver fir and (b) Douglas-fir. The filled circles and the empty circles represent significant and non-significant values of Pearson's correlation coefficients ($P \leq 0.05$), respectively. Each correlation coefficient includes 24 pairs of δ^{18}O-δ^{2}H values (three sites pooled with eight trees each). The dark solid line on each panel shows the fitted relationships following model 8 (Supporting Information). The horizontal lines overlapping each circle show the range of precipitation sum values for the three sites for a given year. Adjusted R^2 and P-values are shown for each fitted model.

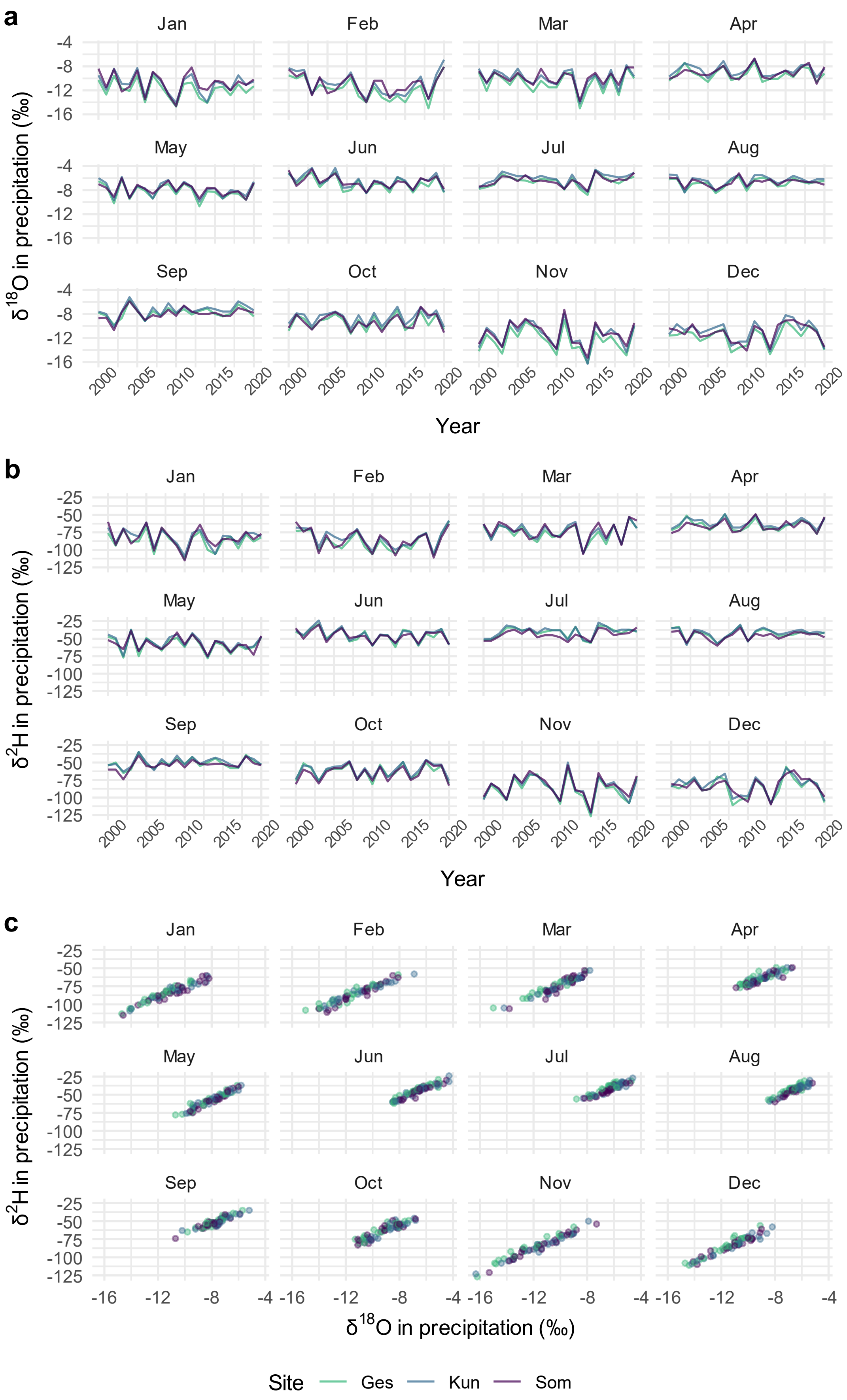

Supplementary Figure 15. Monthly isotope ratios in precipitation with (a) δ^{18}O, (b) δ^{2}H and (c) the δ^{18}O-δ^{2}H relationship. The data were modelled with the tool Piso.AI (see section *2.4. Climate data*).

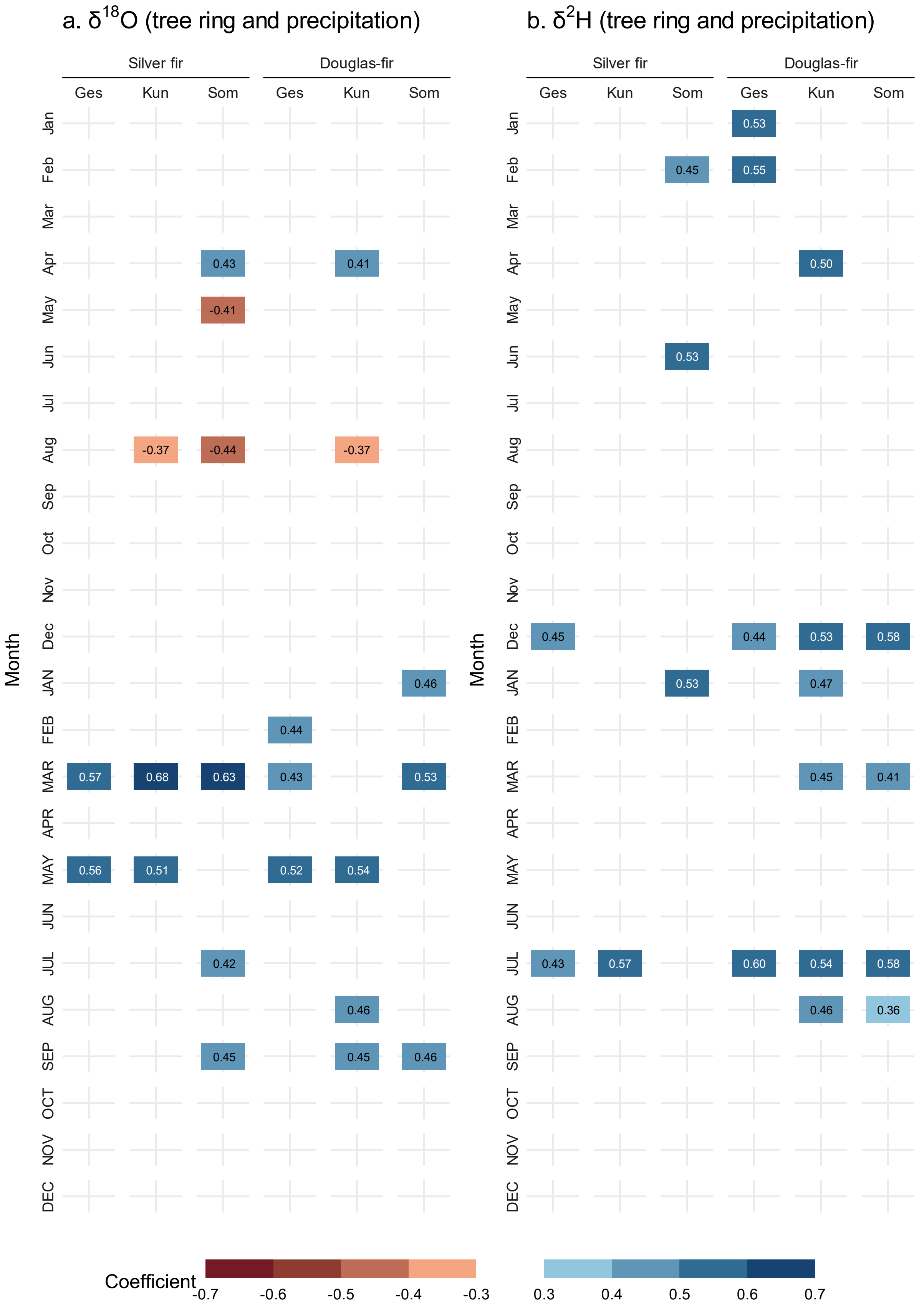

Supplementary Figure 16. Bootstrapped Pearson's correlation coefficients between (a) annual δ^{18}O in tree-ring cellulose and monthly δ^{18}O in precipitation and (b) annual δ^{2}H in tree-ring cellulose and monthly δ^{2}H in precipitation for the period 2000–2020. Months in capital letters indicate months of the current year.

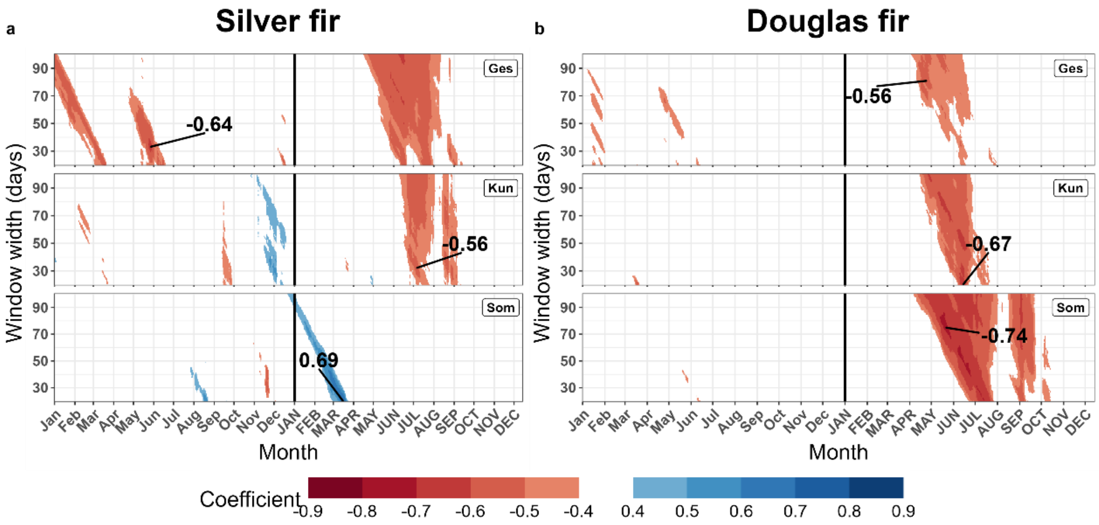

Supplementary Figure 17. Moving-window correlations between daily vapor pressure deficit (VPD) and tree-ring width chronologies of (a) silver fir and (b) Douglas-fir for the period 2000–2020 at the three study sites (labels on the top right corners). Only significant correlations are shown (P-value ≤ 0.05). The day of the month on the x-axis corresponds to the beginning of the window width (e.g., a point at 60 days on the y-axis and the beginning of July on the x-axis corresponds to a correlation with the climate data of 60 days starting at the beginning of July, i.e., covering July and August). Months in lowercase denote the months of the year prior to tree-ring formation. Numbers in bold indicate the highest correlation per site.

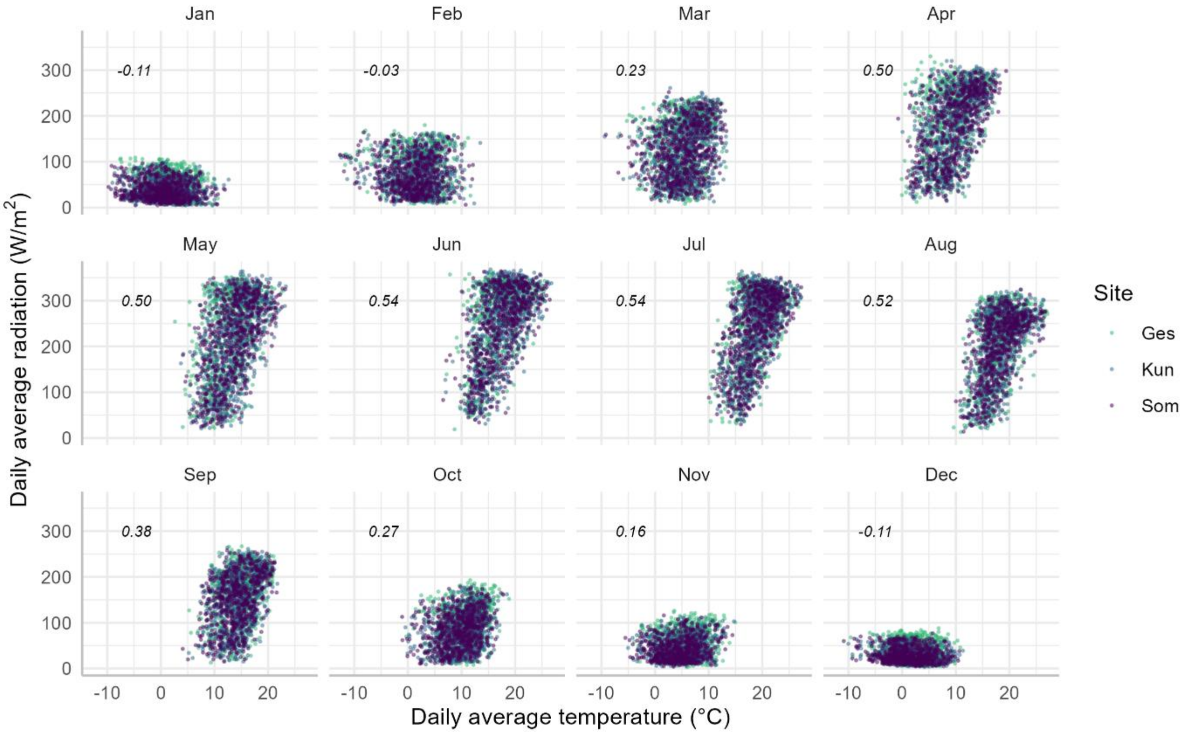

Supplementary Figure 18. Relationship between the daily average global radiation and the daily average temperature per month for the period 1999–2020. The colors represent the three sites. Pearson's correlation coefficients are indicated in the top left corner for each month.

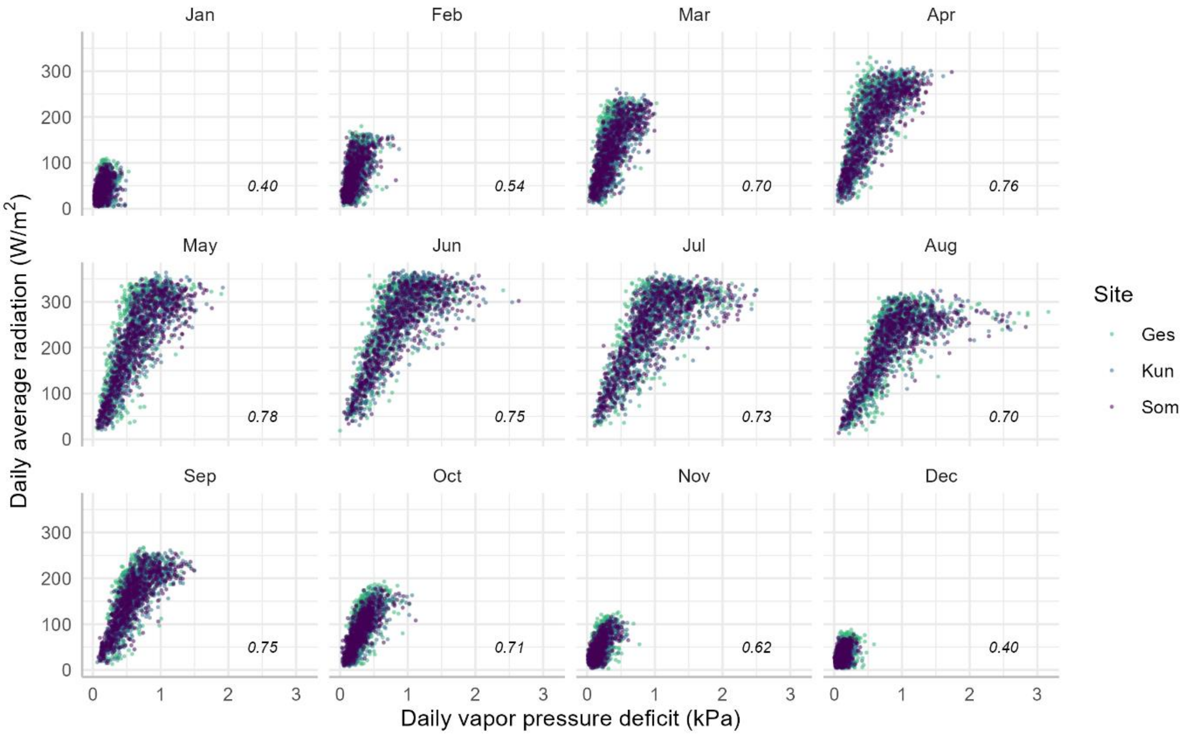

Supplementary Figure 19. Relationship between the daily average global radiation and the daily vapor pressure deficit per month for the period 1999–2020. The colors represent the three sites. Pearson's correlation coefficients are indicated in the bottom right corner for each month.

References

Allen, R. G., Pereira, L. S., Raes, D., Smith, M., & others. (1998). *Crop evapotranspiration-Guidelines for computing crop water requirements-FAO Irrigation and drainage paper 56* (p. 300). FAO – Food and Agriculture Organization of the United Nations.
