# Supplementary material for: Decoupling of Tree‐Ring Cellulose δ 18O and δ 2H Highlighted by Their Contrasting Relationships to Climate and Tree Intrinsic Variables
Source: Plant Cell Environ. 2024 Nov 7;48(3):1903–18. doi: 10.1111/pce.15252 (PMC11788974; doi:10.1111/pce.15252)
Supplement: Supplementary file 2 — Supporting information. [file PCE-48-1903-s001.pdf]

Decoupling of tree-ring cellulose δ^{18}O and δ^{2}H highlighted by their contrasting relationships to climate and tree intrinsic variables

2024-03-25

Description of the statistical models used for the analyses

Relationship d18O-d2H at the individual level (Figure 3a-b)

One model per species (linear mixed-effects model; 2 models)

```
model.1 <- lme(data = data,
        fixed = d2H ~ d18O*TreeID,
        random = ~d18O|SiteID)
```

Relationship d18O-d2H at the site level (Figure 3c-d)

One model per species (linear mixed-effects model; 2 models)

```
model.2 <- lme(data = data,
        fixed = d2H ~ d18O,
        random = ~d18O|SiteID)
```

Relationship d18O/d2H and tree-ring width at the site level (Figure 4)

One model per species and per isotope ratio (additive mixed model; 4 models)

```
model.3 <- gam(data = data,
        REML = T,
        y ~ SiteID +
          s(TRW, by = SiteID, k = 4) +
          s(TreeID, bs = "re")
        )

# y is d18O or d2H
```

Relationship d18O/d2H and tree-ring width at the tree level (Supplementary figures 2, 3, 4 and 5)

One model per species and per isotope ratio (additive mixed model; 4 models)

```
model.4 <- gam(data = data,
        REML = T,
        y ~ TreeID +
          s(TRW, by = TreeID, k = 4) +
          s(SiteID, bs = "re")
       )
```

```
# y is d18O or d2H
```

Relationship d18O/d2H and tree age (Figure 5)

One model per species, per site and per isotope ratio (linear mixed-effects model; 12 models)

```
model.5 <- lme(data = data,
           fixed = y ~ Age,
           random = ~1|TreeID)

# y is d18O or d2H
```

Relationship d18O/d2H and tree crown volume (Figure 6)

One model per species and per isotope ratio (linear mixed-effects model; 4 models)

```
model.6 <- lme(data = data,
           fixed = y ~ Crown_volume,
           random = ~Crown_volume|SiteID)

# y is d18O or d2H
```

Relationship correlation d18O-d2H and VPD summer (Figure 8)

One model per species (linear model; 2 models)

```
model.7 <- lm(data = data,
             cor_d18O_d2H ~ VPD_summer_mean)

# cor_d18O_d2H is the correlation coefficient between d18O and d2H per year (8 trees * 3 sites).
# VPD_summer_mean is the mean of VPD from July to September averaged over the three sites for each year.
```

Relationship correlation d18O-d2H and other climatic variables (Supplementary figures 10, 11, 12, 13 and 14)

One model per species and per climatic parameter (linear model; 10 models)

```
model.8 <- lm(data = data,
             cor_d18O_d2H ~ climatic_variable_mean)

# cor_d18O_d2H is the correlation coefficient between d18O and d2H per year (8 trees * 3 sites).
# climatic_variable_mean is the mean of the climatic variable over the season averaged over the three sites for each year.
# The climatic variable can be VPD, precipitation sum or average temperature.
# The season can be spring (April to June) or summer (July to September).
```
